# Autophagic adaptation to oxidative stress alters peritoneal residential macrophage survival and ovarian cancer metastasis

**DOI:** 10.1172/jci.insight.141115

**Published:** 2020-09-17

**Authors:** Houjun Xia, Shasha Li, Xiong Li, Weichao Wang, Yingjie Bian, Shuang Wei, Sara Grove, Weimin Wang, Linda Vatan, J. Rebecca Liu, Karen McLean, Ramandeep Rattan, Adnan Munkarah, Jun-Lin Guan, Ilona Kryczek, Weiping Zou

**Affiliations:** 1 Department of Surgery,; 2 Center of Excellence for Cancer Immunology and Immunotherapy, and; 3 Department of Obstetrics and Gynecology, University of Michigan School of Medicine, Ann Arbor, Michigan, USA.; 4 Department of Women’s Health Services, Henry Ford Health System, Detroit, Michigan, USA.; 5 Department of Cancer Biology, University of Cincinnati College of Medicine, Cincinnati, Ohio, USA.; 6 Department of Pathology,; 7 Graduate Program in Immunology,; 8 Doctoral Program in Tumor Biology, and; 9 University of Michigan Rogel Cancer Center, University of Michigan School of Medicine, Ann Arbor, Michigan, USA.

**Keywords:** Immunology, Cancer

## Abstract

Tumor-associated macrophages (TAMs) affect cancer progression and therapy. Ovarian carcinoma often metastasizes to the peritoneal cavity. Here, we found 2 peritoneal macrophage subsets in mice bearing ID8 ovarian cancer based on T cell immunoglobulin and mucin domain containing 4 (Tim-4) expression. Tim-4^+^ TAMs were embryonically originated and locally sustained while Tim-4^–^ TAMs were replenished from circulating monocytes. Tim-4^+^ TAMs, but not Tim-4^–^ TAMs, promoted tumor growth in vivo. Relative to Tim-4^–^ TAMs, Tim-4^+^ TAMs manifested high oxidative phosphorylation and adapted mitophagy to alleviate oxidative stress. High levels of arginase-1 in Tim-4^+^ TAMs contributed to potent mitophagy activities via weakened mTORC1 activation due to low arginine resultant from arginase-1–mediated metabolism. Furthermore, genetic deficiency of autophagy element FAK family-interacting protein of 200 kDa resulted in Tim-4^+^ TAM loss via ROS-mediated apoptosis and elevated T cell immunity and ID8 tumor inhibition in vivo. Moreover, human ovarian cancer–associated macrophages positive for complement receptor of the immunoglobulin superfamily (CRIg) were transcriptionally, metabolically, and functionally similar to murine Tim-4^+^ TAMs. Thus, targeting CRIg^+^ (Tim-4^+^) TAMs may potentially treat patients with ovarian cancer with peritoneal metastasis.

## Abstract 2

Ovarian tumor-associated peritoneal macrophages have potent mitochondria activity, which enables reliance on the mitophagy pathway to alleviate oxidative stress and facilitate survival in the tumor microenvironment.

## Graphical abstract

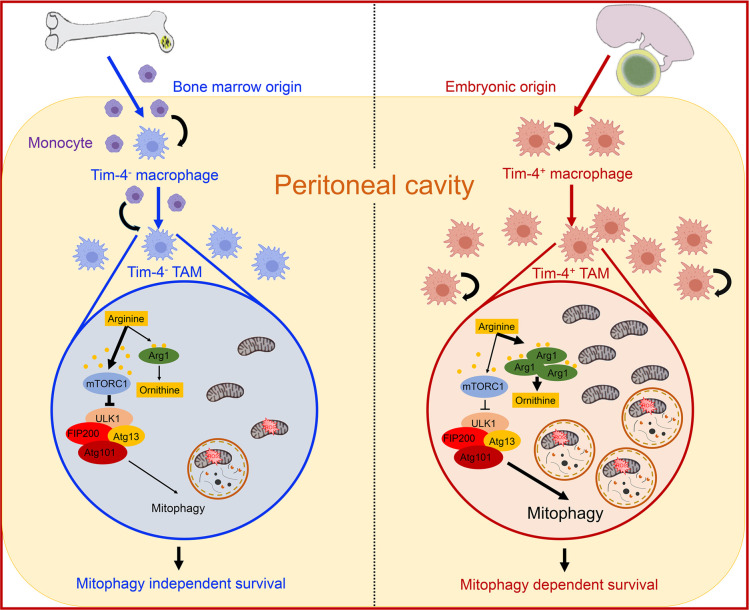

## Introduction

Ovarian cancer frequently metastasizes to the peritoneal cavity as manifested by ascites fluid formation and a large number of tumor islets distributing to the peritoneum, omentum, and serosal surfaces of the viscera. The entire peritoneal cavity becomes an active tumor microenvironment, supporting ovarian cancer metastasis and progression (1). Tumor-associated macrophages (TAMs) constitute over 50% of cells in the peritoneal tumor implants and the ascites fluids in patients with peritoneal ovarian cancer metastasis. Macrophages are physiologically critical mediators of tissue homeostasis. However, TAMs can directly support tumor growth and suppress the tumor immune responses (2–4). In line with this, previous studies in mouse models have shown that peritoneal TAMs generally promoted ovarian cancer metastasis and inhibited immunity (5–7). These observations suggest that TAMs may be an ideal target for cancer immunotherapy (3). Following this thought, different approaches — including targeting TAM trafficking, switching their functions, and developing TAM-depleting antibody — have been tested in preclinical models (8). However, these approaches have failed to translate clinically (9–11). Furthermore, macrophages can uptake, process, and present antigens to T cells and promote antitumor immunity. Macrophages may directly mediate tumor killing and are associated with improved patient outcomes in some types of cancer (12–14). In addition, emerging evidence indicates that monocytes are not the sole and major source of tissue macrophages, and embryonically derived macrophages can form a distinct tissue macrophage subset (15, 16). It seems that embryonically derived macrophages and peripheral monocyte–derived macrophages may play different and often conflicting roles in different types of tumor models (17–19). Therefore, the controversies among the previous studies beg an in-depth understanding of TAM ontogeny, phenotype, metabolism, and functional characteristics in vitro and in vivo, which is critical for eventually developing effective mechanism-informed, TAM-targeted cancer immunotherapy.

In this work, we focus on peritoneal residential macrophages in ovarian cancer models and patients with ovarian cancer. Previous studies have implicated that peritoneal residential macrophages may be distinguished from other tissue residential macrophages in gene profile and function (20–22). Notably, the majority of these studies are realized in steady-state and inflammatory disease models (23, 24). TAM subsets remain poorly understood in ovarian cancer peritoneal metastasis and progression (25). Based on the relative expression levels of F4/80 and MHC-II, previous studies have classified macrophages into 2 subsets, F4/80^hi^MHC-II^lo^ and F4/80^lo^MHC-II^hi^ (5, 26). Here, based on T cell immunoglobulin and mucin domain containing 4 (Tim-4) expression, we identified 2 phenotypically, transcriptionally, ontogenically, metabolically, and functionally distinct TAM subsets, Tim-4^+^ and Tim-4^–^ TAMs, in an ovarian cancer peritoneal metastasis model, then extended our studies to patients with ovarian cancer.

## Results

### Tim-4 defines 2 distinct peritoneal macrophage subsets in ovarian cancer.

**Figure 1:**
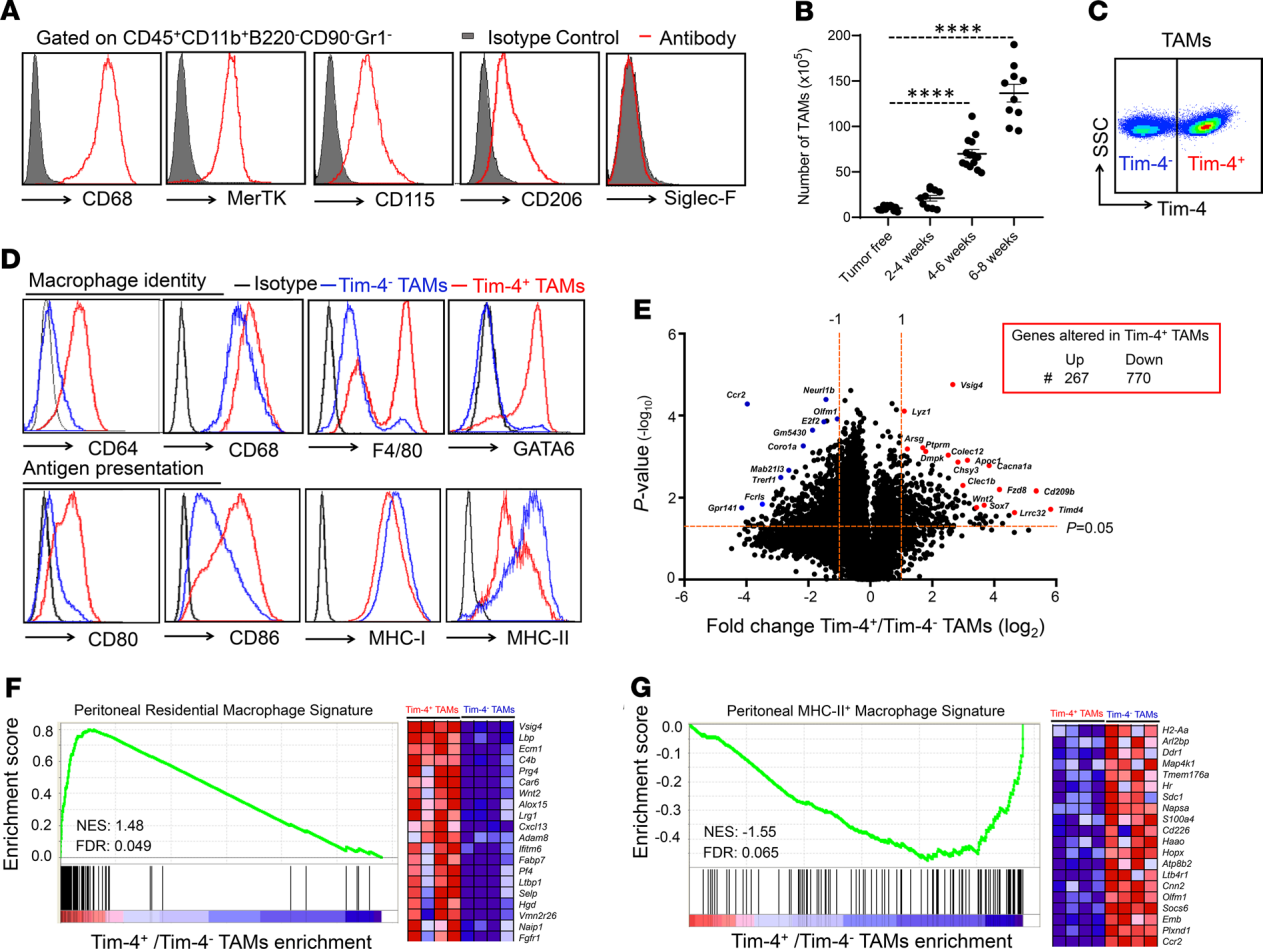
Tim-4 defines 2 distinct peritoneal macrophage subsets in ovarian cancer. (**A**) Measurement of macrophage-related markers on TAMs. CD45^+^CD11b^+^B220^–^CD90^–^Gr1^–^ macrophages were identified in peritoneal single cells in mice bearing peritoneal ID8 ovarian cancer (*n* = 10). (**B**) Dynamic changes of peritoneal TAMs during ID8 peritoneal ovarian cancer progression (*n* = 10–14/group, mean ± SEM). Time points: 2–4, 4–6, and 6–8 weeks. ****P < 0.0001 (1-way ANOVA with Dunnett’s multiple-comparisons test) between tumor-free and tumor-bearing mice. (**C**) Representative surface expression of Tim-4 on peritoneal TAMs. Data are shown at week 4 after ovarian cancer inoculation (*n* = 10). (**D**) Phenotypic difference in Tim-4^+^ and Tim-4^-^ TAMs. TAMs were stained with the indicated antibodies. One representative of 5 is shown. (**E**) Transcripts in TAM subsets. Peritoneal TAM subsets were isolated and sorted from 6 to 7 weeks in ID8 tumor–bearing mice. An RNA-Seq assay was performed in 4 groups of paired Tim-4^+^ and Tim-4^–^ TAMs. Volcano plots show upregulated and downregulated genes based on statistic value *P* < 0.05 and fold change ≥ 2 or ≤ –2. (**F** and **G**) RNA-Seq analysis in TAMs. Positive gene enrichment of residential macrophage gene signatures (**F**) and negative gene enrichment of MHC-II^+^ macrophage gene signatures (**G**) in Tim-4^+^ TAMs compared with Tim-4^–^ TAMs. The 20 most enriched genes are shown on the right side. NES, normalized enrichment score; FDR, false discovery rate. FDR < 0.25 is considered significant.

Ovarian carcinoma often metastasizes to the peritoneal cavity. However, peritoneal residential macrophages are poorly understood in ovarian cancer. To explore the nature of peritoneal TAMs in ovarian cancer, we established ID8 ovarian cancers in the mouse peritoneal cavity and studied TAMs in the peritoneal ascites fluids. Flow cytometry identified CD45^+^CD11b^+^CD90^–^B220^–^Gr1^–^ macrophages in the peritoneal cavity in ID8 tumor–bearing mice (Supplemental Figure 1A; supplemental material available online with this article; https://doi.org/10.1172/jci.insight.141115DS1). These cells expressed multiple macrophage markers, including CD68, CD206, colony-stimulating factor 1 receptor (or CD115), and tyrosine-protein kinase Mer (MerTK), but not Siglec-F, a marker for eosinophils and alveolar macrophages (Figure 1A). The levels of TAMs increased following tumor progression (Figure 1B).

Tim-4 is a marker for embryonically derived residential macrophages in the intestine (27, 28), skin (29), and heart (30). Interestingly, based on Tim-4 expression, we observed 2 distinct peritoneal TAMs: Tim-4^+^ and Tim-4^–^ cells (Figure 1C). To explore whether Tim-4^+^ and Tim-4^–^ peritoneal TAMs were phenotypically different populations, we compared the expression of a panel of macrophage-associated markers on Tim-4^+^ and Tim-4^–^ peritoneal TAMs (Figure 1D). We noticed that Tim-4^+^ TAMs represented the majority of the F4/80^hi^MHC-II^lo^ subset while Tim-4^–^ TAMs represented the majority of the F4/80^lo^MHC-II^hi^ subset (Figure 1D and Supplemental Figure 1B). In addition, Tim-4^+^ and Tim-4^–^ TAMs expressed comparable levels of CD206, PD-L1, and MerTK (Supplemental Figure 1C). Long-lived peritoneal residential macrophages selectively express transcriptional factor GATA6 and its downstream genes (20–22). We found that GATA6 was enriched in Tim-4^+^ TAMs but not Tim-4^–^ TAMs (Figure 1D). Furthermore, Tim-4^+^ TAMs expressed high levels of several GATA6 downstream genes, including *Aspa*, *Cd5l*, *Nt5e*, *Ltbp1*, and *Tgfb2*, as compared with Tim-4^–^ TAMs (20, 22) (Supplemental Figure 1D).

To gain comprehensive insight into the differences between Tim-4^+^ and Tim-4^–^ TAMs, we performed transcriptional profiling on the paired Tim-4^+^ and Tim-4^–^ TAM subsets isolated from ID8 tumor–bearing mice. Among 1037 differentially expressed genes in the paired Tim-4^+^ and Tim-4^–^ TAM subsets, 267 and 770 genes were upregulated and downregulated, respectively, in Tim-4^+^ TAMs as compared with Tim-4^–^ TAMs (Figure 1E). The top upregulated genes, including *Lyz1*, *Vsig4*, and *Wnt2*, were linked to F4/80^hi^MHC-II^–^ residential macrophages (31), while the top downregulated genes, including *Ccr2*, *Cd226*, and *Plxnd1*, were associated with MHC-II^hi^ monocyte-derived macrophages (Supplemental Table 1) (32). Gene set enrichment analysis (GSEA) revealed an enriched F4/80^hi^MHC-II^–^ residential macrophage gene signature (Figure 1F) (31) and a weak MHC-II^+^ monocyte-derived macrophage gene signature (Figure 1G) (32) in Tim-4^+^ TAMs as compared with Tim-4^–^ TAMs. Together, the data suggest that Tim-4 identifies 2 phenotypically distinct peritoneal macrophage subsets in ovarian cancer. We next tested whether Tim-4^+^ and Tim-4^–^ peritoneal TAMs are ontogenically, metabolically, and functionally distinct subsets.

### Tim-4^–^ TAMs migrate from peripheral monocytes without affecting tumor growth.

**Figure 2:**
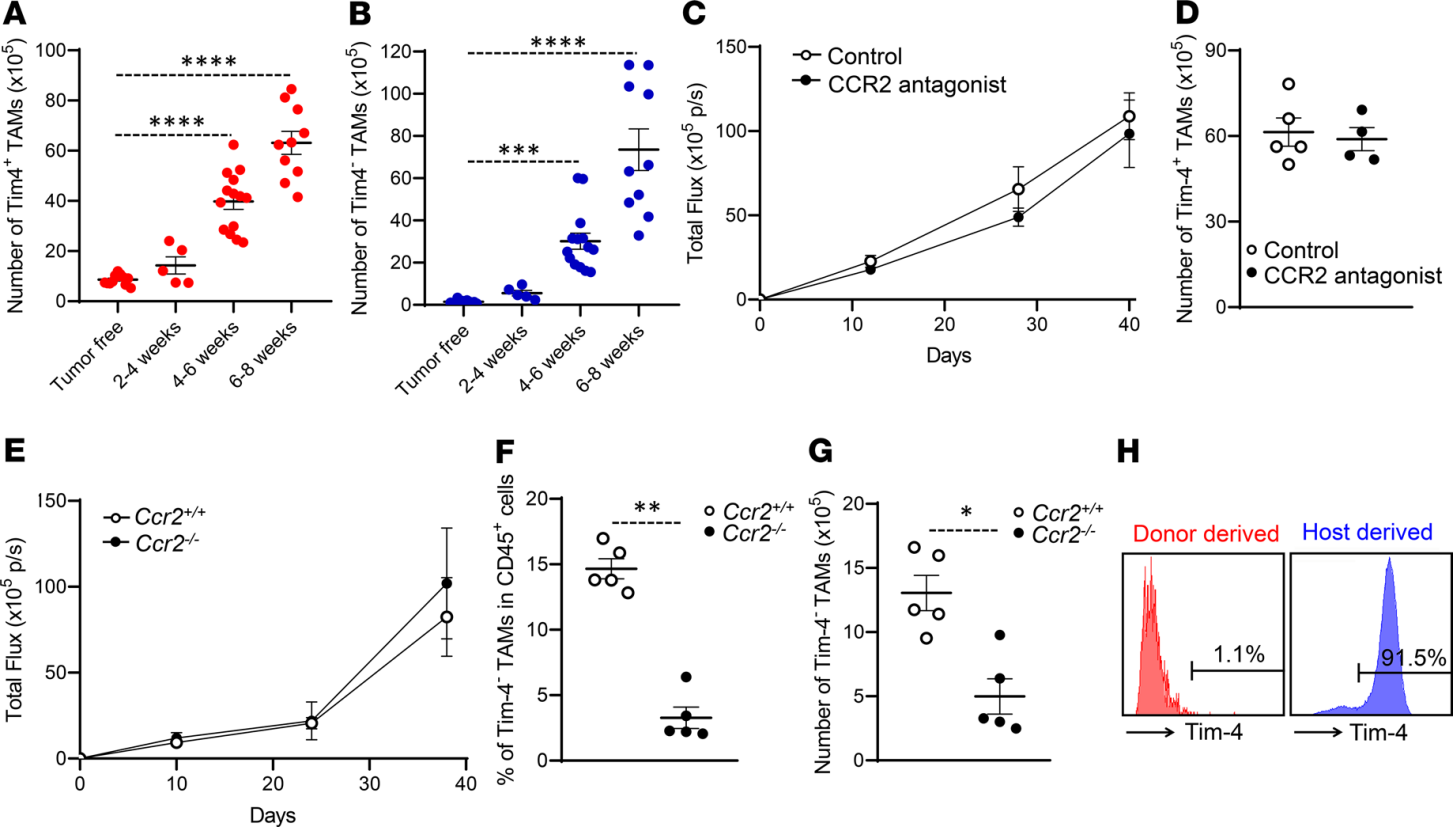
Tim-4^–^ TAMs migrate from peripheral monocytes without affecting tumor growth. (**A** and **B**) Dynamic changes of Tim-4^+^ (**A**) and Tim-4^–^ (**B**) TAM numbers during peritoneal ovarian cancer progression (*n* = 5 to 14 mice/group, mean ± SEM). ***P < 0.001, ****P < 0.0001 (1-way ANOVA with Dunnett’s multiple-comparisons test) between tumor-free and tumor-bearing mice at different time points. (**C**) Tumor growth in mice treated with CCR2 antagonist or PBS (*n* = 8 to 10 mice/group, mean ± SEM). (**D**) Tim-4^+^ TAM numbers in mice treated with CCR2 antagonist or PBS (*n* = 4 to 5 mice/group, mean ± SEM). (**E**) ID8 tumor growth in *Ccr2^+/+^* and *Ccr2^–/–^* mice (*n* = 5 mice/group, mean ± SEM). (**F** and **G**) Effect of CCR2 deficiency on Tim-4^–^ TAMs. Percentage (**F**) and number (**G**) of Tim-4^–^ TAMs were compared between *Ccr2^+/+^* and *Ccr2^–/–^* mice bearing ID8 tumors. (*n* = 5 mice/group, mean ± SEM). *P < 0.05, **P < 0.01 (Mann-Whitney *U* test). (**H**) Source of Tim-4^+^ TAMs. CD45.1^+^ monocytes were transferred (I.P.) into ID8 tumor–bearing CD45.2 mice. Tim-4 was determined on CD45.1^+^ (shown in red) and CD45.2^+^ (shown in blue) TAMs. One representative of 4 is shown. p/s, photons/second.

Based on our phenotypic and transcriptional profile on Tim-4^+^ and Tim-4^–^ TAMs, we hypothesized that Tim-4^+^ TAMs were embryonically derived residential macrophages and Tim-4^–^ TAMs were replenished from blood monocytes. In healthy mice (tumor free), Tim-4^+^ and Tim-4^–^ peritoneal macrophages, respectively, accounted for 85% and 15% of peritoneal macrophages in homeostasis (Supplemental Figure 2, A and B). After tumor inoculation, absolute numbers of Tim-4^+^ (Figure 2A) and Tim-4^–^ (Figure 2B) TAMs increased following tumor progression. Interestingly, the percentage of Tim-4^+^ TAMs in TAMs gradually shrank (Supplemental Figure 2A), while the percentage of Tim-4^–^ TAMs in TAMs moderately increased (Supplemental Figure 2B). Six to 8 weeks after tumor inoculation, there were approximately 50% each of Tim-4^+^ (Figure 2A) and Tim-4^–^ (Figure 2B) TAMs in mice.

Next, we explored the origin of Tim-4^+^ and Tim-4^–^ TAMs in 3 confirmatory settings. CCR2 mediates peripheral monocyte trafficking into the peritoneal cavity (32, 33). The first experimental setting was a pharmacological depletion model. We treated adult mice with sc-202525, a CCR2 antagonist, for 2 weeks. Treatment with sc-202525 resulted in reduced F4/80^lo^MHC-II^hi^ peritoneal macrophages and had no effect on F4/80^hi^MHC-II^lo^ macrophages (Supplemental Figure 2C) (33). Then, we inoculated ID8 tumor cells into the peritoneal cavity in these pretreated mice and treated them with sc-202525 continually for 6 weeks. We found comparable tumor volume (Figure 2C) and a similar quantity of Tim-4^+^ TAMs (Figure 2D) in mice receiving PBS and CCR2 antagonist. These data suggest that blockade of monocyte peritoneal trafficking has no obvious effect on Tim-4^+^ TAMs and ID8 tumor growth.

The second experimental setting was a genetic model. We compared peritoneal macrophages in CCR2-deficient (*Ccr2*^–/–^) and -sufficient (*Ccr2*^+/+^) mice. We observed a similar number of total peritoneal macrophages in tumor-free *Ccr2*^–/–^ and *Ccr2*^+/+^ mice (Supplemental Figure 2D). However, the percentage (Supplemental Figure 2E) and absolute number (Supplemental Figure 2F) of Tim-4^–^, but not Tim-4^+^, peritoneal macrophages were reduced in *Ccr2*^–/–^ mice. We inoculated ID8 tumor cells into the peritoneal cavity of *Ccr2*^+/+^ and *Ccr2*^–/–^ mice. Again, we observed similar tumor growth (Figure 2E) in *Ccr2*^–/–^ and *Ccr2*^+/+^ mice. As expected, Tim-4^–^ TAMs remained limited in *Ccr2*^–/–^ tumor-bearing mice as compared with *Ccr2*^+/+^ mice (Figure 2, F and G). These data suggest that monocyte trafficking deficiency has no obvious effect on ID8 tumor growth.

The third experimental setting was an adoptive immune cell transfusion system. We explored whether Tim-4^+^ TAMs could be directly differentiated from monocytes in the tumor microenvironment. We injected CD45.1^+^ monocytes into ID8 tumor–bearing cognate CD45.2 mice. Three days later, we observed that CD45.1^+^ donor TAMs were in the F4/80^lo^MHC-II^hi^ population (Supplemental Figure 2G) and expressed no Tim-4 (Figure 2H). Together, these data suggest that circulating monocytes are a cellular source for Tim-4^–^ TAMs, and Tim-4^+^ TAMs may be embryonic residential macrophages.

### Tim-4^+^ TAMs are embryonically derived proliferative cells with protumor function.

**Figure 3:**
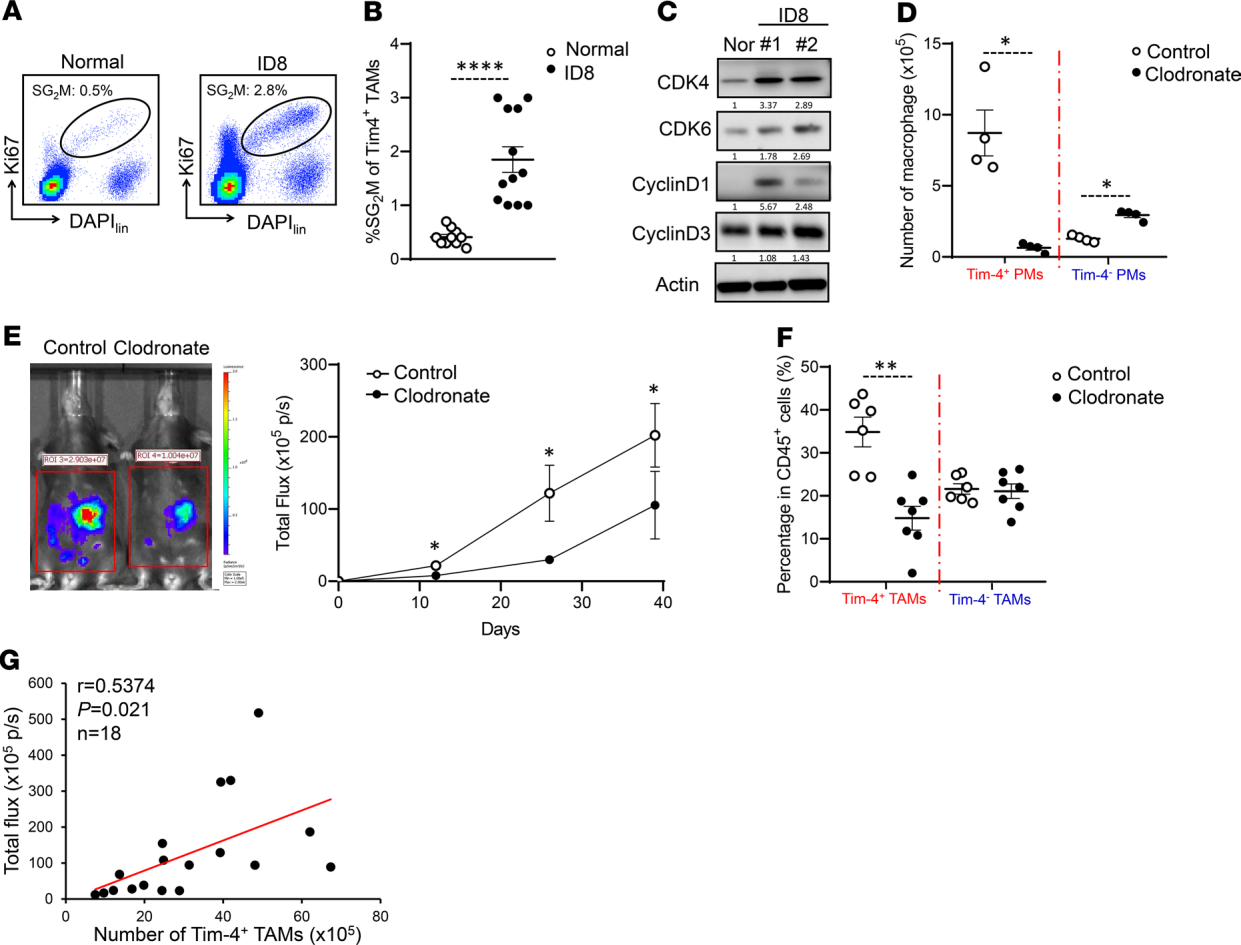
Tim-4^+^ TAMs are embryonically derived proliferative cells with protumor function. (**A** and **B**) Proliferative status of Tim-4^+^ TAMs in tumor-bearing mice. (**A**) Flow cytometry dot plots represent the percentage of Tim-4^+^ macrophages in SG_2_M phase. (**B**) Percentage of SG_2_M-positive macrophages in Tim-4^+^ peritoneal macrophages from normal and ID8 tumor–bearing mice (*n* = 10–12 mice/group, mean ± SEM). ****P < 0.0001 (Mann-Whitney *U* test). (**C**) Western blot showing cell cycle proteins in Tim-4^+^ peritoneal residential macrophages and Tim-4^+^ TAMs. One representative of 3 is shown. (**D**) Effect of CLs on peritoneal macrophages (PMs). Tim-4^+^ and Tim-4^–^ peritoneal macrophage numbers were quantified in tumor-free mice treated with control liposome or CL (*n* = 4 mice/group, mean ± SEM). *P < 0.05 (Mann-Whitney *U* test) between control and clodronate-treated mice in both Tim-4^+^ PMs and Tim-4^–^ PMs. (**E**) Tumor growth between control liposome– and CL-pretreated mice (*n* = 6–8 mice/group, mean ± SEM). *P < 0.05 (Mann-Whitney *U* test) between control and clodronate-treated mice at days 12, 26, and 39. (**F**) Percentage of TAM subsets in total CD45^+^ immune cells between control and clodronate treatment (*n* = 6–7 mice/group, mean ± SEM). **P < 0.01 (Mann-Whitney *U* test) between control and clodronate-treated mice. (**G**) The Pearson correlation between Tim-4^+^ TAM numbers and tumor load. Red line indicates regression fit.

Both Tim-4^+^ and Tim-4^–^ TAMs were increased following tumor progression. Tim-4^–^ TAMs were from peripheral monocytes. We hypothesized that increased Tim-4^+^ TAM numbers are attributed to their local self-expansion in the tumor microenvironment. To test this, we compared the proliferative capacity of Tim-4^+^ macrophages in normal and tumor-bearing mice (21, 34). As expected, Tim-4^+^ TAMs exhibited increased cell proliferation as shown by more TAMs in the SG_2_M phase, when compared with normal Tim-4^+^ residential macrophages (Figure 3, A and B). As a confirmation, we injected BrdU into the peritoneal cavity in tumor-bearing mice for 3 hours and observed increased BrdU^+^ Tim-4^+^ TAMs (Supplemental Figure 3, A and B). Accompanied with this, we detected elevated expression of cell cycle regulator transcripts (Supplemental Figure 3C) and proteins (Figure 3C). Thus, Tim-4^+^ TAMs are locally proliferative residential macrophages.

As there is no direct approach to specifically manipulate Tim-4^+^ residential macrophages in vivo, we applied 2 complementary models to explore a potential role of Tim-4^+^ TAMs in tumor growth. In the first model, we mixed ID8 tumor cells and Tim-4^+^ TAMs isolated from ID8-bearing mice and inoculated WT mice with the mixture. We found that addition of Tim-4^+^ TAMs promoted tumor growth (Supplemental Figure 3D). In the second model, we used a modified method to deplete Tim-4^+^ macrophages with clodronate liposomes (CLs) (6, 19). We initially treated normal mice with 1 dose of CLs, followed by a 14-day chase period to allow mice to recover circulating monocytes (Supplemental Figure 3E). We found that circulating monocyte numbers were similar in CL-treated and control mice at 14 days following CL treatment (Supplemental Figure 3F), and the levels of Tim-4^+^ macrophages were significantly lower in CL-treated mice than control mice (Figure 3D). Then, we peritoneally inoculated these mice with ID8 tumor cells. Under this condition, we observed a slower tumor growth in CL-treated mice as compared with control mice (Figure 3E). On day 42, the percentage of Tim-4^+^ TAMs remained lower in CL-treated mice than control mice, while the percentage of Tim-4^–^ TAMs was comparable in the CL-treated mice and control mice (Figure 3F). Furthermore, the number of Tim-4^+^ TAMs positively correlated with tumor volume in tumor-bearing mice (Figure 3G). Altogether, the data suggest that Tim-4^+^ TAMs are embryonically derived proliferative cells and promote tumor growth.

### Tim-4^+^ TAMs exhibit and maintain high mitochondria activity and mitophagy function.

**Figure 4:**
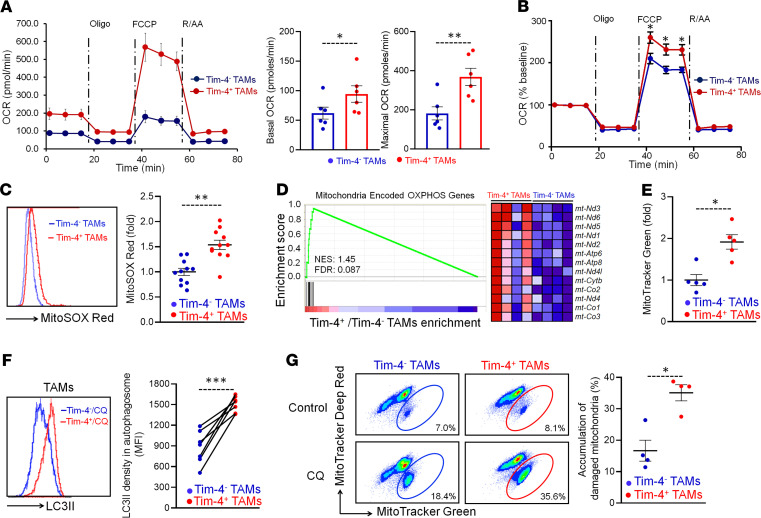
Tim-4^+^ TAMs exhibit and maintain high mitochondria activity and autophagy function. (**A** and **B**) Oxygen consumption rates (OCRs) in TAM subsets. Peritoneal TAM subsets were sorted from ID8 tumor–bearing mice and treated with oligomycin (Oligo), FCCP, and rotenone plus antimycin A (R/AA). (**A**) OCR was determined by using the Seahorse XF Extracellular Flux Analyzer in Tim-4^+^ and Tim-4^–^ TAMs. Basal and maximal respiration was shown. (*n* = 6 mice/group, mean ± SEM). *P < 0.05, **P < 0.01 (paired Student’s *t* test). (**B**) Percentage of OCR was normalized to basal level in Tim-4^+^ TAMs and Tim-4^–^ TAMs. (*n* = 6 mice/group, mean ± SEM). *P < 0.05 (Mann-Whitney *U* test) between Tim-4^+^ TAMs and Tim-4^–^ TAMs at the indicated time point. (**C**) Measurement of mitochondria related ROS in Tim-4^+^ and Tim-4^–^ TAMs (*n* = 11 mice/group, mean ± SEM). **P < 0.01 (Mann-Whitney *U* test). (**D**) Positive enrichment of the mitochondrial DNA–encoded, OXPHOS-related genes in Tim-4^+^ TAMs compared with Tim-4^–^ TAMs. Enrichment score plots (left panel); 13 enriched genes (right panel). FDR < 0.25 is considered significant. (**E**) Mitochondrial mass of Tim-4^+^ and Tim-4^–^ TAMs (*n* = 5 mice/group, mean ± SEM). *P < 0.05 (Mann-Whitney *U* test). (**F**) Measurement of LC-3II density in autophagosome between Tim-4^+^ and Tim-4^–^ TAMs (*n* = 7 mice/group). The paired Tim-4^+^ and Tim-4^–^ TAMs were from the same mice. ***P < 0.001 (Mann-Whitney *U* test). (**G**) Flow cytometry dot plots showing the percentage of damaged mitochondria in TAM subsets treated with or without CQ for 24 hours. The difference between CQ treatment and control was considered as accumulated damaged mitochondria in Tim-4^+^ TAMs and Tim-4^–^ TAMs. (*n* = 4 mice/group, mean ± SEM). *P < 0.05 (Mann-Whitney *U* test).

Metabolism affects immune cell phenotype and function in the tumor microenvironment (35). Elevated oxidative phosphorylation is observed in ID8 and B16 tumor–associated peritoneal macrophages as compared with normal peritoneal macrophages in mice (6). Given that Tim-4^+^ and Tim-4^–^ TAMs are phenotypically and functionally different, we hypothesized that peritoneal TAM subsets may present different mitochondria activities. To test this, we carried out Seahorse experiments to assess and compare the functional profile of mitochondria in Tim-4^+^ and Tim-4^–^ TAMs. TAMs were treated with oligomycin (an ATP synthase inhibitor), cyanide p-trifluoromethoxyphenyl-hydrazone (FCCP) (H^+^ ionophore), and rotenone plus antimycin A (inhibitors of the electron transport chain) (Figure 4A). We observed higher levels of basal and maximal respiration (Figure 4A) in Tim-4^+^ TAMs than Tim-4^–^ TAMs, as shown by the OCR. After normalizing the OCR values to baseline, Tim-4^+^ TAMs continued to exhibit an elevated maximal respiration as compared with Tim-4^–^ TAMs (Figure 4B). The data suggest that Tim-4^+^ TAMs manifest high mitochondria activity. In line with this, we observed higher levels of mitochondria-related ROS (Figure 4C) in Tim-4^+^ TAMs compared with Tim-4^–^ TAMs. In further support of this, the mitochondrial genome–encoded, oxidative phosphorylation–related genes were highly enriched in Tim-4^+^ TAMs but not Tim-4^–^ TAMs (Figure 4D). MitoTracker Green staining showed enhanced mitochondrial mass in Tim-4^+^ TAMs compared with Tim-4^–^ TAMs (Figure 4E). Western blot revealed elevated expression of mitochondria-related proteins COX IV and SDHB in Tim-4^+^ TAMs compared with Tim-4^–^ TAMs (Supplemental Figure 4A).

Oxidative stress is a bystander metabolic feature associated with oxidative phosphorylation (OXPHOS). Dendritic cells (36) and regulatory T cells (37) are phenotypically and functionally altered by oxidative stress in the tumor microenvironment. Autophagy alleviates oxidative stress by eliminating damaged mitochondria and ROS in a process called mitophagy (38). To investigate if autophagy activity was different in TAM subsets in response to high OXPHOS, we treated macrophages with chloroquine (CQ), an autophagy inhibitor, to prevent autophagosome degradation. Flow cytometry analysis revealed an increased LC-3II density in autophagosomes in Tim-4^+^ TAMs compared with Tim-4^–^ TAMs (Figure 4F). LC-3II is the lipidated form of LC-3 specifically located in the autophagosome. Western blot demonstrated a high conversion of LC-3I to LC-3II in Tim-4^+^ TAMs compared with Tim-4^–^ TAMs (Supplemental Figure 4B). To evaluate the effect of autophagy on mitochondria fitness in Tim-4^+^ and Tim-4^–^ TAMs, we treated TAMs with CQ for 24 hours and detected damaged mitochondria accumulation via a combination of MitoTracker Green, a membrane potential–independent (ΔΨm-independent) mitochondrial stain, and MitoTracker Deep Red, a ΔΨm-dependent mitochondrial stain (39). As expected, treatment with CQ caused more accumulation of damaged mitochondria (MitoTracker Green^hi^, MitoTracker Deep Red^lo^) in Tim-4^+^ TAMs compared with that in Tim-4^–^ TAMs (Figure 4G). The data suggest that Tim-4^+^ TAMs highly rely on mitophagy to maintain mitochondrial fitness in the tumor microenvironment.

### Arginase-1 affects mitochondria fitness and mitophagy via mTORC1 in Tim-4^+^ TAMs.

**Figure 5:**
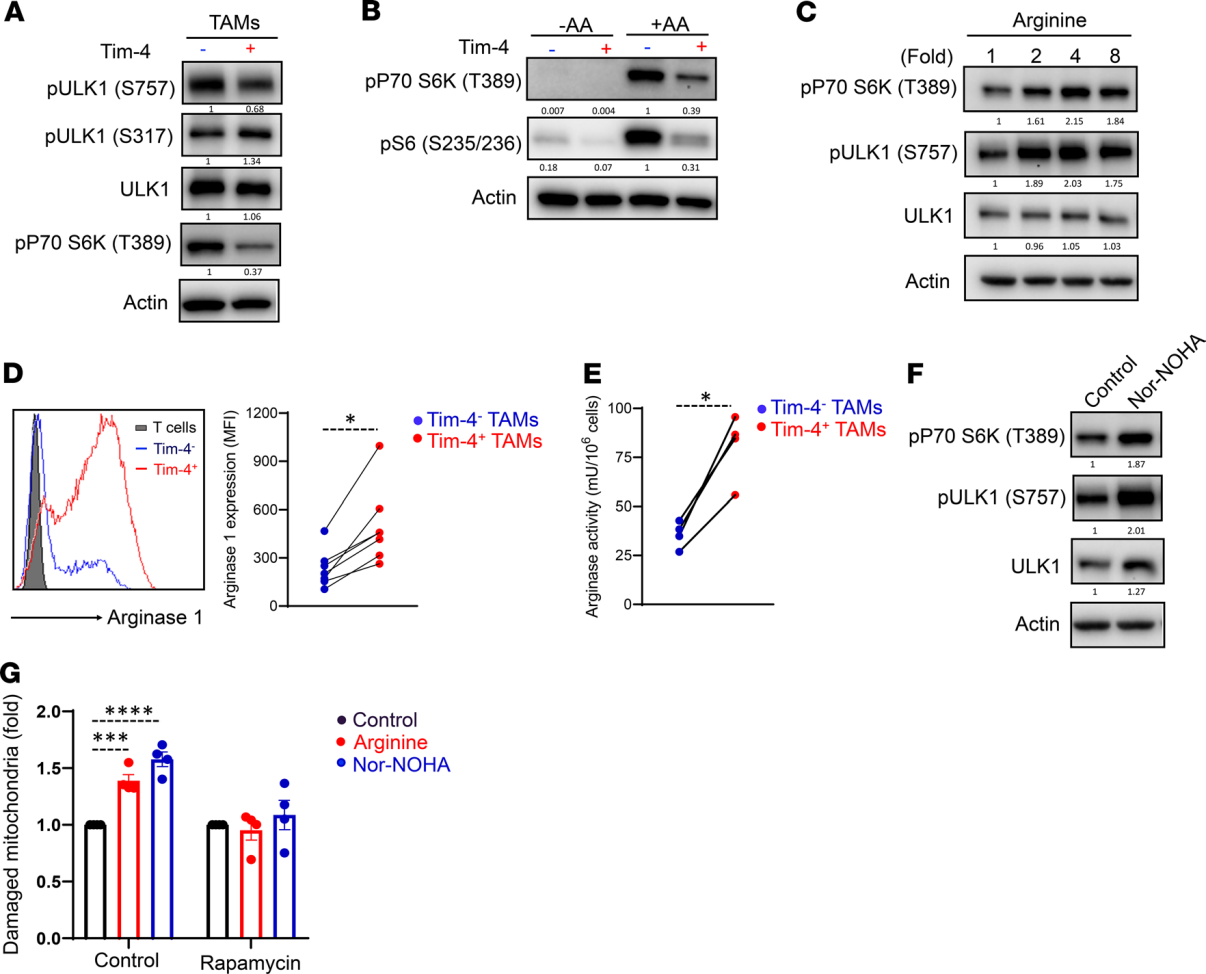
Arginase-1 affects mitochondria fitness and mitophagy via mTORC1 in Tim-4^+^ TAMs. (**A**) Western blot showing mTORC1 activity in fresh Tim-4^+^ TAMs and Tim-4^–^ TAMs. One representative of 3 is shown. (**B**) Western blot showing mTORC1 activity in Tim-4^+^ TAMs and Tim-4^–^ TAMs. TAMs were isolated from tumor-bearing mice and cultured overnight in completed medium. TAMs were stimulated in amino acid–free (-AA) medium or full amino acid medium (+AA) for 4 hours. One representative of 3 is shown. (**C**) Western blot showing mTORC1 activity in Tim-4^+^ TAMs treated with arginine for 24 hours. One representative of 3 is shown. (**D**) Measurement of arginase-1 expression in Tim-4^+^ TAMs and Tim-4^–^ TAMs by flow cytometry. The paired Tim-4^+^ and Tim-4^–^ TAMs were from the same mouse. (*n* = 7 mice/group). *P < 0.05 (Mann-Whitney *U* test). (**E**) Measurement of arginase activity Tim-4^+^ TAMs and Tim-4^–^ TAMs by ELISA. The paired Tim-4^+^ and Tim-4^–^ TAMs were from the same mice. (*n* = 4 mice/group). *P < 0.05 (Mann-Whitney *U* test). (**F**) Western blot showing mTORC1 activity in Tim-4^+^ TAMs treated with arginase inhibitor nor-NOHA (0.5 mM), for 24 hours. One representative of 3 is shown. (**G**) Effect of arginine on damaged mitochondria accumulation (mitophagy) in Tim-4^+^ TAMs. In the absence or presence of rapamycin, mitochondria damage was induced as described in Tim-4^+^ TAMs without (control), or with, arginine or arginase-1 inhibitor nor-NOHA. Damaged mitochondria accumulation was determined by FACS. Results were normalized to control. (*n* = 4 mice/group, mean ± SEM). ***P < 0.001, ****P < 0.0001 (1-way ANOVA with Dunnett’s multiple-comparisons test).

We next examined the mechanism by which Tim-4^+^ TAMs exhibited high mitophagy activity. GSEA revealed no enrichment of mitophagy-related genes in Tim-4^+^ TAMs compared with Tim-4^–^ TAMs (Supplemental Figure 5A). This suggests that mitophagy-related autophagy genes may be regulated at posttranscriptional levels in Tim-4^+^ and Tim-4^–^ TAMs. Unc-51-like kinase 1 (ULK1) phosphorylation at Ser757 by mammalian target of rapamycin complex 1 (mTORC1) competes with phosphorylation at Ser317 by AMPK to inhibit the initiation of the canonical autophagy pathway (40). As compared with Tim-4^–^ TAMs, we detected lower levels of ULK1 phosphorylation at Ser757 and similar levels of ULK1 phosphorylation at Ser317 in Tim-4^+^ TAMs (Figure 5A). We suspected that mTORC1 activity may be reduced in Tim-4^+^ TAMs. As expected, Tim-4^+^ TAMs manifested a weak mTORC1 activity as compared with Tim-4^+^ TAMs, as shown by low levels of phosphorylation of S6 kinase (Figure 5A), a well-characterized mTORC1 substrate (41). Amino acid sensing may determine mTORC1 activation (42). We explored a potential role of amino acids on mTORC1 activity in TAM subsets. We cultured TAM subsets in amino acid–free medium and observed a potent inhibition of mTORC1 activation in both Tim-4^+^ and Tim-4^–^ TAMs. Interestingly, supplementation of amino acids induced a robust mTORC1 activation in Tim-4^–^ TAMs but not in Tim-4^+^ TAMs (Figure 5B). Intracellular arginine can sustain mTORC1 activation in regulatory T cells (43). To evaluate whether arginine is involved in maintaining mTORC1 activation in Tim-4^+^ TAMs, we treated Tim-4^+^ TAMs with different concentrations of arginine. As expected, exogenous arginine could induce and maintain mTORC1 activation in Tim-4^+^ TAMs (Figure 5C). Thus, arginine, as a key amino acid, might define different mTORC1 activities in Tim-4^+^ and Tim-4^–^ TAMs.

Intracellular arginine is controlled by arginine uptake and metabolism. We detected comparable levels of arginine uptake in Tim-4^+^ TAMs and Tim-4^–^ TAMs (Supplemental Figure 5B). Arginase-1 converts intracellular arginine to downstream metabolites, including ornithine and urea. Western blot revealed high levels of arginase-1 in total TAMs compared with normal peritoneal macrophages (Supplemental Figure 5C) (44). RNA-Seq data showed high levels of arginase-1 transcripts in Tim-4^+^ TAMs compared with Tim-4^–^ TAMs (Supplemental Figure 5D), and comparable expression levels of other genes in polyamine biogenesis, including *Odc1* and *Srm*, in Tim-4^–^ and Tim-4^+^ TAMs (Supplemental Figure 5D). In line with this, flow cytometry analysis (Figure 5D) and Western blot (Supplemental Figure 5E) demonstrated superior levels of arginase-1 protein in Tim-4^+^ TAMs compared with Tim-4^–^ TAMs. Notably, arginase-2 was not detectable in both TAM subsets (Supplemental Figure 5E). In addition, we detected higher levels of arginase activity in Tim-4^+^ TAMs than in Tim-4^–^ TAMs (Figure 5E). Then, we treated Tim-4^+^ TAMs with *N*-hydroxy-nor-arginine (nor-NOHA), an arginase activity inhibitor, to block the arginine consumption. We observed that nor-NOHA stimulated mTORC1 activation in Tim-4^+^ TAMs (Figure 5F). Hence, weak mTORC1 activation may be attributed to high levels of arginase-1 and low levels of intracellular arginine in Tim-4^+^ TAMs. To further elucidate a role of arginine in mitochondrial fitness and mitophagy in Tim-4^+^ TAMs, we treated Tim-4^+^ TAMs with a combination of mitochondrial inhibitors oligomycin and antimycin A, which collapse ΔΨm (45), in the presence of arginine or nor-NOHA. Oligomycin and antimycin A combination increased damaged mitochondria in Tim-4^+^ TAMs (Supplemental Figure 5F). Interestingly, treatment with arginine or nor-NOHA resulted in an increase in damaged mitochondria in Tim-4^+^ TAMs, and this effect was abolished with rapamycin (Figure 5G). The data suggest that intracellular arginine mediates mitophagy inhibition via mTORC1 activity in Tim-4^+^ TAMs. Thus, arginase-1 fine-tunes mitochondria fitness and mitophagy in Tim-4^+^ TAMs.

### Autophagy deficiency results in loss of Tim-4^+^ TAMs in ovarian cancer.

**Figure 6:**
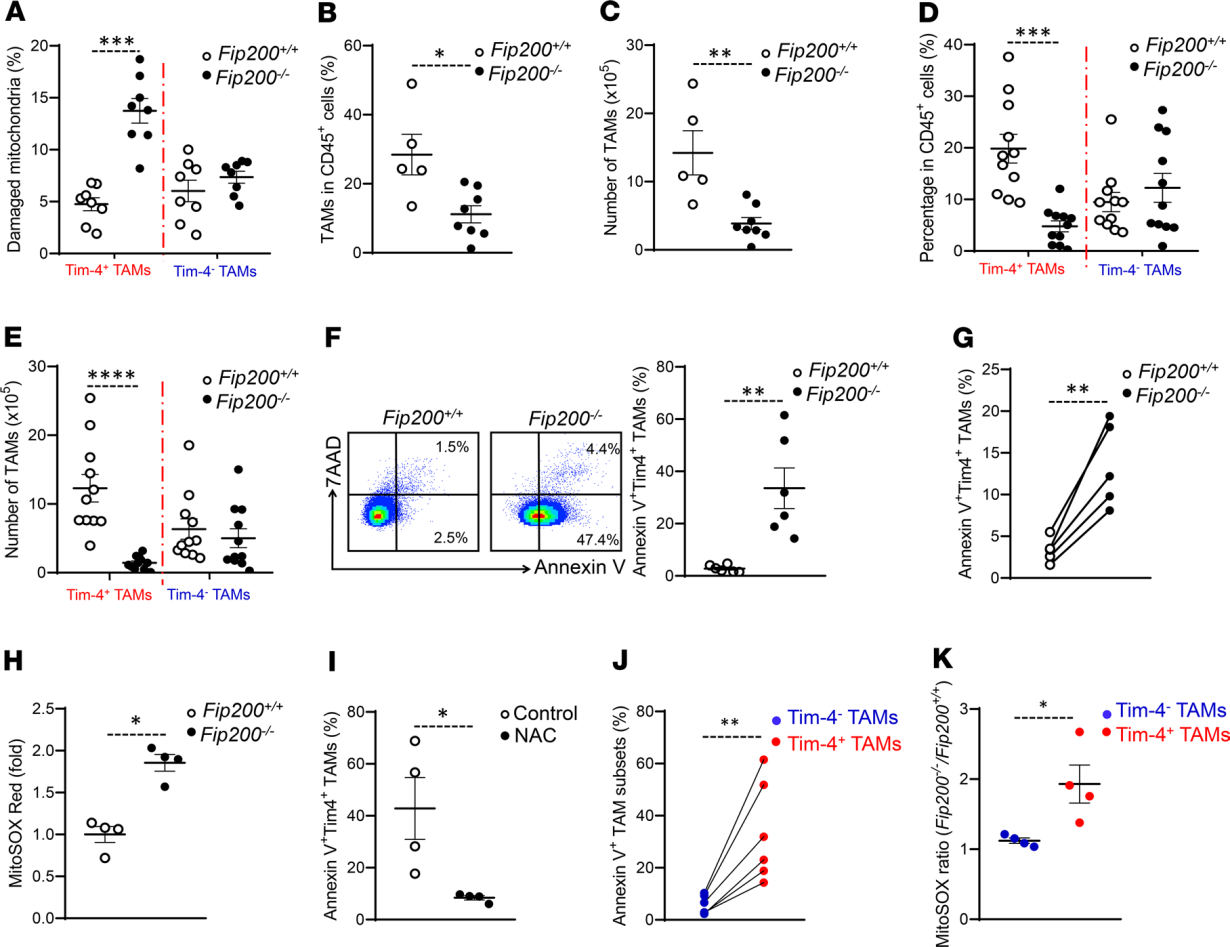
Autophagy deficiency results in loss of Tim-4^+^ TAMs in ovarian cancer. (**A**) Effect of FIP200 deficiency on mitochondria in Tim-4^+^ TAMs and Tim-4^–^ TAMs. The percentage of Tim-4^+^ TAMs and Tim-4^–^ TAMs with damaged mitochondria in *Fip200^+/+^* and *Fip200^–/–^* tumor-bearing mice. (*n* = 8 mice/group, mean ± SEM.) ***P < 0.001 (Mann-Whitney *U* test). (**B** and **C**) Percentage (**B**) and number (**C**) of TAMs in total immune cells between *Fip200^+/+^* and *Fip200^–/–^* tumor-bearing mice (*n* = 5 to 8 mice/group, mean ± SEM). *P < 0.05, **P < 0.01 (Mann-Whitney *U* test). (**D** and **E**) Percentage (**D**) and number (**E**) of Tim-4^+^ and Tim-4^–^ TAM subsets in total immune cells between *Fip200^+/+^* and *Fip200^–/–^* tumor-bearing mice (*n* = 11 mice/group, mean ± SEM). ***P < 0.001, ****P < 0.0001 (Mann-Whitney *U* test) in Tim-4^+^ TAMs between *Fip200^+/+^* and *Fip200^–/–^*. (**F**) Apoptosis of Tim-4^+^ TAMs in *Fip200^+/+^* and *Fip200^–/–^* tumor-bearing mice. (*n* = 6 mice/group, mean ± SEM). **P < 0.01 (Mann-Whitney *U* test). (**G**) Effect of FIP200 deficiency on Tim-4^+^ TAM apoptosis. TAMs were analyzed for apoptosis 36 days after tumor inoculation (*n* = 5 mice/group). **P < 0.01 (Mann-Whitney *U* test). (**H**) Measurement of mitochondria-related ROS in Tim-4^+^ TAMs between *Fip200^+/+^* and *Fip200^–/–^* tumor-bearing mice (*n* = 4 mice/group, mean ± SEM). *P < 0.05 (Mann-Whitney *U* test). (**I**) Effect of NAC on Tim-4^+^ TAM apoptosis in vivo (*n* = 4 mice/group, mean ± SEM). *P < 0.05 (Mann-Whitney *U* test). (**J**) Comparison of apoptosis between paired Tim-4^+^ and Tim-4^–^ TAMs in *Fip200^–/–^* tumor-bearing mice (*n* = 6 mice/group). **P < 0.01 between Tim-4^+^ versus Tim-4^–^ TAMs in *Fip200^–/–^* tumor-bearing mice (Mann-Whitney *U* test). (**K**) Mitochondrial ROS ratio of *Fip200^–/–^* versus *Fip200^+/+^* in TAM subsets (*n* = 4 mice/group, mean ± SEM). *P < 0.05 (Mann-Whitney *U* test).

Mitophagy degrades damaged mitochondria and removes ROS to prevent macrophage death (46). High levels of mitophagy in Tim-4^+^ TAMs might be important to support their survival in the tumor microenvironment. To test this possibility in vivo, we established ID8 tumor mouse models with autophagy deficiency in macrophages. FAK family-interacting protein of 200 kDa (FIP200) is one component of the ULK1-Atg13-FIP200-Atg101 complex and is essential for the induction of mammalian autophagy (47). We crossed mice with transgenic expression of Cre recombinase from the *Lysozyme* promoter with *loxP*-flanked FIP200 alleles (*Fip200^fl/fl^*) mice and deleted the *loxP*-flanked FIP200 alleles specifically in myeloid cells (called *Fip200^–/–^* here). Western blot showed a specific loss of FIP200 and an increase in autophagy receptor p62 in macrophages isolated from the peritoneal cavity and differentiated from bone marrow of *Fip200^–/–^* mice (Supplemental Figure 6A). We inoculated *Fip200^–/–^* mice with ID8 tumor cells and detected high levels of damaged mitochondria accumulated in Tim-4^+^ *Fip200^–/–^* TAMs but not in Tim-4^–^ *Fip200^–/–^* TAMs compared with their WT counterparts. The data additionally support the notion that Tim-4^+^ TAMs, but not Tim-4^–^ TAMs, relied on mitophagy in the tumor microenvironment in vivo (Figure 6A). To functionally understand if mitophagy is important to Tim-4^+^ TAMs’ survival, we analyzed the impact of autophagy deficiency in TAM numbers and phenotype in tumor-bearing mice. We found that the percentage (Figure 6B) and number (Figure 6C) of total TAMs was decreased in *Fip200^–/–^* mice as compared with control mice. Interestingly, we observed a loss of Tim-4^+^ TAMs, but not Tim-4^–^ TAMs, in ID8 bearing *Fip200^–/–^* mice compared with the WT mice, as shown by the percentage (Figure 6D) and absolute number (Figure 6E) of Tim-4^+^ and Tim-4^–^ TAMs. To explore whether loss of Tim-4^+^ TAMs is attributed to the tumor microenvironment, we analyzed peritoneal residential macrophage subsets in tumor-free mice. We found comparable numbers of Tim-4^+^ residential macrophages in WT and *Fip200^–/–^* mice (Supplemental Figure 6B). The results suggest that loss of Tim-4^+^ TAMs in *Fip200^–/–^* mice is related to tumor challenge.

Tim-4^+^ macrophages are of embryonic origin. Tim-4^+^ TAM pool may be determined by the balance between self-expansion and survival. Interestingly, we observed an increase in SG_2_M percentage in Tim-4^+^ TAMs in *Fip200^–/–^* mice compared with WT mice. This indicates that loss of Tim-4^+^ TAMs is unlikely due to a proliferative defect (Supplemental Figure 6C). T cell survival is impaired in the tumor microenvironment due to metabolic challenge (48, 49). We analyzed TAM death and survival. We observed increased annexin V (Figure 6F) and cleaved caspase-3 expression (Supplemental Figure 6D) in *Fip200*^–/–^ Tim-4^+^ TAMs as compared with WT cells. To understand if cell death is associated with intrinsic factor, we isolated CD45.2^+^Tim-4^+^ residential macrophages from *Fip200^–/–^* mice and injected them into the peritoneal cavity in WT CD45.1^+^ mice, and inoculated these mice with ID8 cells. We found 3- to 5-fold more apoptosis in *Fip200*-deficient CD45.2^+^Tim-4^+^ TAMs compared with WT CD45.1^+^Tim-4^+^ TAMs (Figure 6G and Supplemental Figure 6E). These data suggest that *Fip200* deficiency results in Tim-4^+^ TAMs’ death in the tumor microenvironment.

GATA6 may control peritoneal residential macrophage development and survival (20–22). We questioned if autophagy deficiency downregulated GATA6 expression in Tim-4^+^ TAMs and affected their development in tumor. Expression of GATA6 and downstream genes was similar in WT and Tim-4^+^ *Fip200^–/–^* TAMs (Supplemental Figure 6F). Given that damaged mitochondria accumulated in Tim-4^+^ TAMs in *Fip200^–/–^* mice compared with WT mice (Figure 6A), we hypothesized increased mitochondria-related ROS may be responsible for Tim-4^+^ TAM death. Indeed, we observed increased mitochondria-related ROS (Figure 6H) and DNA damage marker pH2A.X (Supplemental Figure 6G) in Tim-4^+^ TAMs from *Fip200^–/–^* mice compared with WT mice. In addition, we treated the *Fip200^–/–^* tumor-bearing mice with ROS scavenger *N*-acetylcysteine (NAC) and analyzed Tim-4^+^ TAM apoptosis. NAC treatment improved survival of autophagy-deficient Tim-4^+^ TAMs (Figure 6I). To further understand if mitophagy supports TAMs’ survival by eliminating ROS in the tumor microenvironment, we compared the levels of cell apoptosis and ROS production between Tim-4^+^ *Fip200^–/–^* TAMs and Tim-4^–^ *Fip200^–/–^* TAMs because they showed different levels of damaged mitochondria accumulation (Figure 6A). We found a dramatic increase of apoptosis in *Fip200*-deficient Tim-4^+^ TAMs as compared with *Fip200*-deficient Tim-4^–^ TAMs (Figure 6J). In line with this, ROS accumulation was elevated in Tim-4^+^ *Fip200^–/–^* TAMs but not in Tim-4^–^ *Fip200^–/–^* TAMs (Figure 6K). Thus, autophagy deficiency causes a loss of Tim-4^+^ TAMs via accumulated ROS in the ovarian cancer microenvironment.

### Autophagy deficiency in macrophages supports T cell–mediated antitumor immunity.

**Figure 7:**
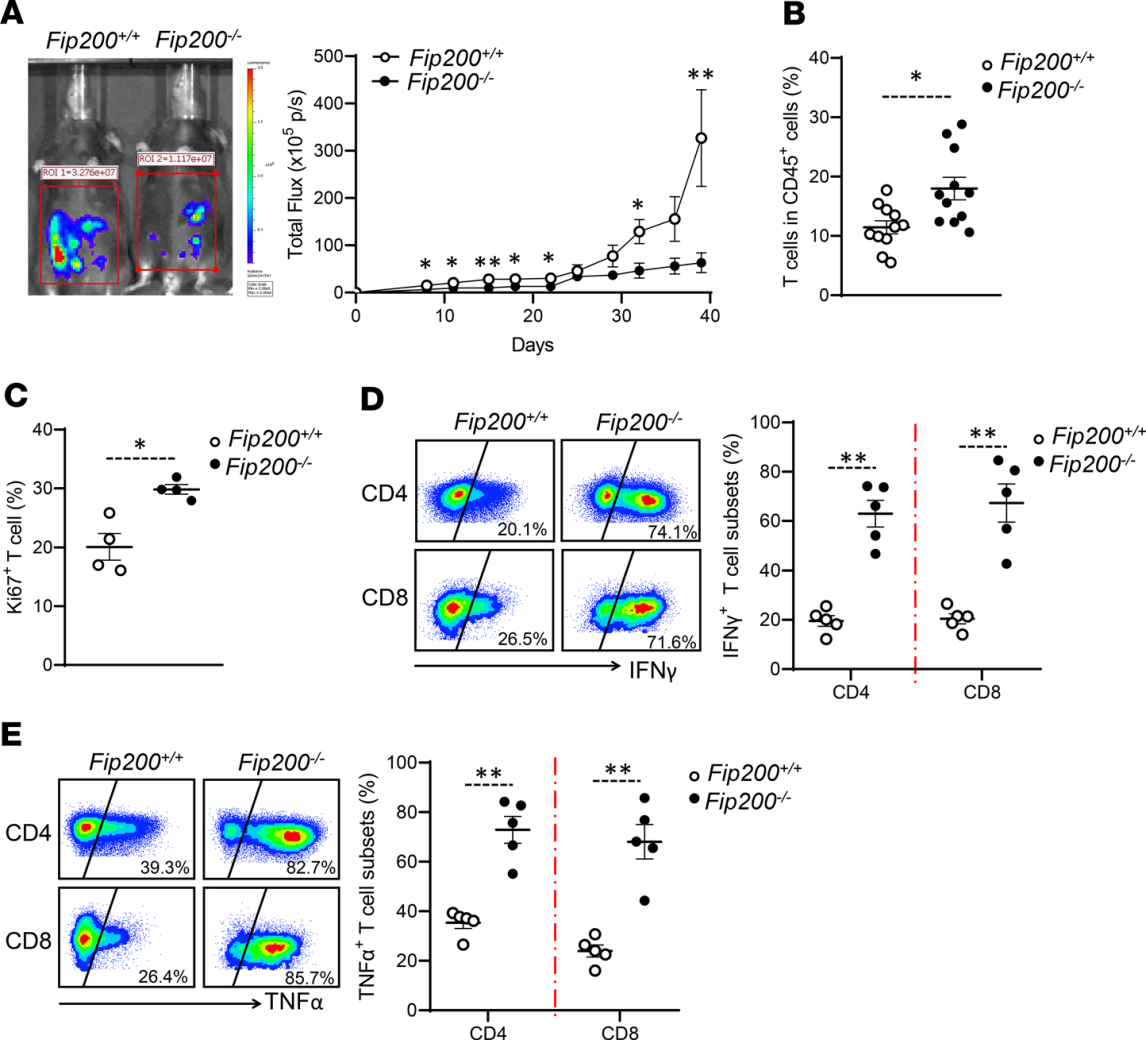
Autophagy deficiency in macrophages supports T cell–mediated antitumor immunity. (**A**) Effect of FIP200 deficiency on peritoneal ovarian cancer progression. WT (*Fip200^+/+^*) and FIP200-deficient (*Fip200^–/–^*) mice were (I.P.) injected with ID8 tumor cells. Tumor growth was monitored (*n* = 6 to 8 mice/group, mean ± SEM). *P < 0.05, **P < 0.01 (Mann-Whitney *U* test) between *Fip200^+/+^* and *Fip200^–/–^* tumor-bearing mice. (**B**) Percentage of T cells in total immune cells between *Fip200^+/+^* and *Fip200^–/–^* tumor-bearing mice (*n* = 11 mice/group, mean ± SEM). *P < 0.05 (Mann-Whitney *U* test). (**C**) Percentage of Ki67^+^ T cells between *Fip200^+/+^* and *Fip200^–/–^* tumor-bearing mice (*n* = 4 mice/group, mean ± SEM). *P < 0.05 (Mann-Whitney *U* test). (**D** and **E**) Percentage of IFN-γ^+^ (**D**) and TNF-α^+^ (**E**) T cell subsets in *Fip200^+/+^* and *Fip200^–/–^* tumor-bearing mice (*n* = 5 mice/group, mean ± SEM). **P < 0.01 (Mann-Whitney *U* test) in both CD4^+^ and CD8^+^ T cells.

Given Tim-4^+^ TAMs, but not Tim-4^–^ TAMs, contributed to ovarian cancer growth (Figure 2 and Figure 3), we studied whether loss of Tim-4^+^ TAMs affects ovarian cancer progression in autophagy-deficient mice. We found that FIP200 deficiency in macrophages slowed down tumor growth compared with WT mice (Figure 7A). In addition, we inoculated MC38 colon cancer cells into the peritoneal cavity of *Fip200^–/–^* mice. Again, FIP200 deficiency in myeloid cells resulted in a slower tumor growth compared with WT mice (Supplemental Figure 7). Furthermore, we analyzed T cell phenotype and cytokine profile in ID8 models. We detected an increase in the percentage of tumor-infiltrating CD90^+^ T cells (Figure 7B) and Ki67^+^ T cells (Figure 7C) in *Fip200*^–/–^ mice compared with WT mice. Furthermore, we detected higher levels of IFN-γ (Figure 7D) and TNF-α (Figure 7E) expression in tumor-infiltrating CD4^+^ and CD8^+^ T cells in *Fip200^–/–^* tumor-bearing mice compared with WT tumor-bearing mice. Thus, autophagy deficiency in macrophages supports T cell–mediated antitumor immunity and slowed down tumor progression.

### TAMs positive for complement receptor of the immunoglobulin superfamily are the human counterparts of murine Tim-4^+^ TAMs in ovarian cancer.

**Figure 8:**
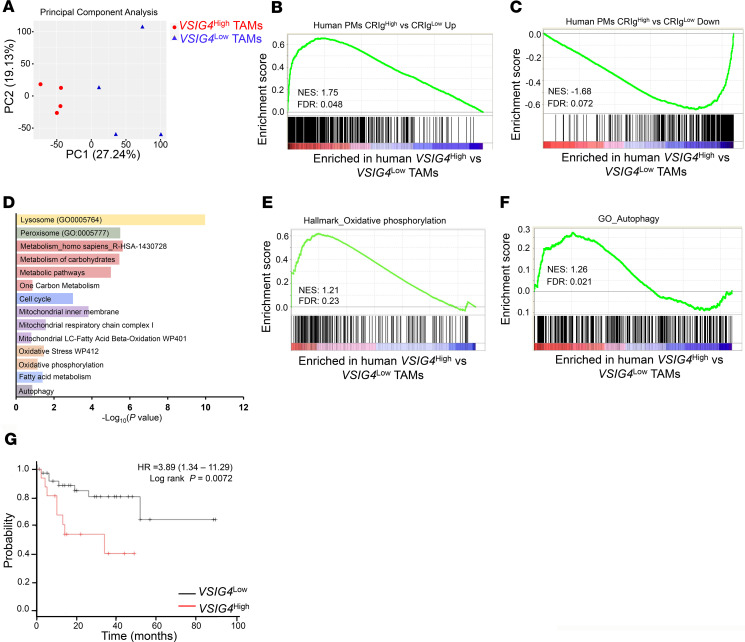
CRIg^+^ TAMs are the murine counterparts of Tim-4^+^ TAMs in human ovarian cancer. (**A**–**F**) Signaling pathway analysis in human ovarian cancer TAM subsets. Based on *VSIG4* transcript levels in the RNA-Seq data set (25), ovarian cancer ascites TAMs were divided into *VSIG4*^hi^ and *VSIG4*^lo^ groups. The *VSIG4*^hi^ group included top 4 higher *VSIG4* expressing TAMs (sample#: TAM43, TAM69, TAM72, and TAM105), while the *VSIG4*^lo^ group included bottom 4 lower *VSIG4* expressing TAMs (sample#: TAM31, TAM92, TAM108, and TAM117). (**A**) Principal components analysis plot of 13,778 genes expressed in *VSIG4*^hi^ and *VSIG4*^lo^ TAMs. (**B** and **C**) Gene set enrichment in human CRIg^hi^ macrophages and *VSIG4*^hi^ TAMs. The significantly upregulated or downregulated genes (FC ≥ 2 or ≤ –2, *P* < 0.05) were determined in human CRIg^hi^ macrophages as compared with CRIg^lo^ macrophages, and in *VSIG4*^hi^ TAMs as compared with *VSIG4*^lo^ TAMs. Positive enrichment of upregulated gene set in human CRIg^hi^ macrophages and *VSIG4*^hi^ TAMs is shown (**B**). Negative enrichment of downregulated gene set in human CRIg^hi^ macrophage and *VSIG4*^hi^ TAMs is shown (**C**). (**D**–**F**) Enrichment of several pathways (**D**), OXPHOS gene (**E**), and autophagy gene (**F**) sets in *VSIG4*^hi^ TAMs compared with *VSIG4*^lo^ TAMs. FDR < 0.25 is considered significant. (**G**) Ovarian cancer patients were divided into high (16 patients) and low (39 patients) *VSIG4* expression groups. Patient survival was shown based on *VSIG4* transcript levels. FDR < 0.25 is considered significant.

In an effort to find the human equivalent of mouse Tim-4^+^ TAMs, we examined Tim-4 expression in TAMs in patients with ovarian cancer. Flow cytometry analysis identified CD45^+^CD3^–^CD14^+^ TAMs in primary ovarian tumor tissues, metastatic omentum, and ascites fluids in patients. We detected 3% of Tim-4^+^ TAMs with a commercially available monoclonal anti–human Tim-4 antibody in the human ovarian cancer microenvironment (Supplemental Figure 8A). The data suggest 2 possibilities: This anti–human Tim-4 clone may not be sensitive to detect membrane Tim-4 on human residential TAMs in ovarian cancer. Or, Tim-4 may not be an operational marker for human residential TAMs in ovarian cancer. To explore potential markers for human ovarian cancer residential TAMs with similar features to mouse Tim-4^+^ TAMs, we assessed genes encoding transmembrane receptors in mouse Tim-4^+^ TAMs. V-set and immunoglobulin domain containing 4 (*Vsig4*) was among the most enriched genes in mouse Tim-4^+^ TAMs compared with Tim-4^–^ TAMs (Figure 1E). *Vsig4* encodes a protein that is also known as a complement receptor of the immunoglobulin superfamily (CRIg) (50). We found that the vast majority of CRIg^+^ TAMs were Tim-4^+^ TAMs in ID8 tumor–bearing mice (Supplemental Figure 8B). FACS analysis identified CRIg^+^ TAMs in patients with ovarian cancer (Supplemental Figure 8C). In addition, *VSIG4* transcripts were highly correlated with the expression of the alternative macrophage marker *CD163* in human ovarian cancer (Supplemental Figure 8D) (51). In line with our FACS data, TAMs highly expressed *VSIG4* transcripts in human ovarian cancer (25). We wondered if human CRIg^+^ TAMs could share genetic and biological features of mouse Tim-4^+^ TAMs. We first compared human ovarian TAM transcriptomes with the established signature of mouse Tim-4^+^ and Tim-4^–^ TAMs (Figure 1E) by using the connectivity MAP (cMAP) (52), which generates scores (as scaled, dimensionless quantities) indicative of the degree of “closeness” of one cell subset to a defined signature gene set. Based on *VSIG4* transcript levels from the RNA-Seq data in human ovarian cancer TAMs (25), cMAP analysis revealed that *VSIG4*^hi^ and *VSIG4*^lo^ human TAMs aligned closely with the gene expression patterns of mouse Tim-4^+^ and Tim-4^–^ TAMs, respectively (Supplemental Figure 8E). In addition, principal components analysis could segregate the transcriptomic profiles of *VSIG4*^hi^ TAMs from *VSIG4*^lo^ TAMs (Figure 8A). It is reported that CRIg^hi^ macrophages may represent peritoneal residential macrophages in humans as compared with CRIg^lo^ macrophages (53). In line with this, GSEA revealed that CRIg^hi^ peritoneal macrophages shared enriched upregulated genes (Figure 8B) and downregulated genes (Figure 8C) with *VSIG4*^hi^ TAMs (53). Furthermore, *VSIG4*^hi^ TAMs expressed enriched genes for lysosome organization and formation (Supplemental Figure 8F), while *VSIG4*^lo^ TAMs were highly enriched for genes for inflammatory response (Supplemental Figure 8G). The data suggest that, at the transcriptional level, *VSIG4*^hi^ TAMs represent CRIg^hi^ residential macrophages in human ovarian cancer ascites.

To additionally characterize *VSIG4*^hi^ TAMs in human ovarian cancer, we used the Functional Annotation Clustering tool in Enrichr to identify significantly enriched pathways and gene ontologies in *VSIG4*^hi^ TAMs. Based on the significantly upregulated genes, metabolic pathways and mitochondria activity were strongly enriched in *VSIG4*^hi^ TAMs as compared with *VSIG4*^lo^ TAMs (Figure 8D). Particularly, *VSIG4*^hi^ TAMs were enriched with an OXPHOS gene set compared with *VSIG4*^lo^ TAMs (Figure 8E). The data suggest that *VSIG4*^hi^ residential TAMs may go through high levels of OXPHOS. In line with this, we observed more mitochondrial mass and high mitochondria-related ROS in CRIg^+^ TAMs compared with CRIg^–^ TAMs in human peritoneal ovarian cancer tissues (Supplemental Figure 8H). In addition, we observed that *VSIG4*^hi^ TAMs were significantly enriched with autophagy-associated gene transcripts compared with *VSIG4*^lo^ TAMs (Figure 8F), which might be due to the higher arginase-1 activity in CRIg^+^ TAMs compared with CRIg^–^ TAMs (Supplemental Figure 8I). Furthermore, high mRNA levels of *VSIG4* were associated with poor survival (Figure 8G) in patients with ovarian cancer. Altogether, the results suggest that human ovarian cancer CRIg^+^ TAMs may be transcriptionally, metabolically, and functionally similar to the equivalent mouse Tim-4^+^ TAMs.

## Discussion

Peritoneal residential macrophage subsets are poorly understood in ovarian cancer. Mouse macrophages are traditionally classified into 2 subsets: F4/80^hi^MHC-II^lo^ and F4/80^lo^MHC-II^hi^. This classification is arbitrarily based on the relative expression levels of F4/80 and MHC-II. Moreover, the expression levels of F4/80 and MHC-II are subject to environmental regulation. Hence, this classification does not serve in sorting specific TAM subsets to study their nature at the transcriptional, metabolic, and functional levels. To meet this challenge, using murine peritoneal ovarian cancer as a model, we found that Tim-4 expression explicitly defines 2 TAM subsets in the ovarian cancer–bearing peritoneal cavity: Tim-4^+^ and Tim-4^–^ cells. Interestingly, Tim-4^+^ and Tim-4^–^ TAMs are respectively enriched in F4/80^hi^MHC-II^lo^ and F4/80^lo^MHC-II^hi^ cells (31, 32). It has been reported that F4/80^hi^MHC-II^lo^ and F4/80^lo^MHC-II^hi^ cells are embryonically originated and are peripheral monocyte–derived cells, respectively (32, 33). Analogously, our complementary and confirmatory experiments reveal that Tim-4^+^ TAMs are embryonically originated and locally proliferative cells, while Tim-4^–^ TAMs are replenished from circulating monocytes. In further support of these findings, it has been reported that Tim-4 marks certain tissue residential macrophages, including heart, intestine, skin, and peritoneal cavity (21, 28, 30, 33, 54). Therefore, Tim-4 is a reasonable phenotypic marker to define TAM subsets in ovarian cancer.

We next explore a potential functional difference between Tim-4^+^ and Tim-4^–^ TAMs in mouse ovarian cancer. The CCL2 and CCR2 signaling pathway mediates peripheral monocyte trafficking into the tumor microenvironment (55). In line with this, we have observed that the levels of peritoneal Tim-4^–^ TAMs (monocyte-derived TAMs) are reduced in *Ccr2*-KO mice and in mice treated with CCR2 antagonist, as compared with controls. However, the reduction of Tim-4^–^ TAMs did not affect peritoneal tumor progression in either *Ccr2*-KO mice or CCR2 antagonist–treated mice. In contrast, we found that deletion of Tim-4^+^ TAMs (embryo-derived TAMs) results in reduced peritoneal tumor progression. In line with this, a recent study has reported a protumor role of omentum CD163^+^Tim-4^+^ TAMs in metastatic ovarian cancer (56). Thus, we suggest that embryo-derived TAMs (Tim-4^+^) and peripheral monocyte–derived TAMs (Tim-4^–^) are biologically different in peritoneal ovarian cancer progression, and specifically targeting Tim-4^+^ (but not Tim-4^–^) TAMs may be therapeutically beneficial to control ovarian cancer and/or other types of cancer peritoneal metastasis and progression.

In an effort to additionally characterize Tim-4^+^ and Tim-4^+^ TAMs, we have examined their major metabolic profiles. Compared with Tim-4^–^ TAMs, Tim-4^+^ TAMs presented higher levels of OXPHOS, higher levels of all mitochondrial DNA–encoded OXPHOS-related genes, and greater mitochondria mass and ROS production. These data indicate that Tim-4^+^ TAMs have experienced high levels of oxidative stress in the tumor microenvironment. In line with this possibility, Tim-4^+^ TAMs expressed high levels of active autophagy. To explore the relevance of autophagy activation in Tim-4^+^ TAMs, we genetically deleted autophagy gene FIP200 in myeloid cells. We observed that FIP200 deficiency abrogated peritoneal ovarian cancer progression and improved T cell–mediated antitumor immunity. Accompanied with this, we noticed a loss of Tim-4^+^ TAMs in FIP200-deficient mice bearing peritoneal ovarian cancer, but not Tim-4^–^ TAMs. This reinforces a protumor role of Tim-4^+^ TAMs. In support of this, FIP200 deficiency selectively results in high apoptosis of Tim-4^+^ TAMs due to accumulation of damaged mitochondria and mitochondria-related ROS. The data suggest that Tim-4^+^ TAMs, but not Tim-4^–^ TAMs, rely on mitophagy to survive in the tumor microenvironment. Interestingly, we found that Tim-4^+^ TAMs express high levels of arginase-1 and efficiently convert arginine to downstream metabolites, resulting in limited intracellular arginine. Sensing of amino acids, including arginine, causes mTORC1 activation in immune cells (57). In line with this, we have detected low levels of mTORC1 activity in Tim-4^+^ TAMs. As mTORC1 is a negative regulator of classical autophagy through phosphorylating ULK1 (40), this may explain high mitophagy in Tim-4^+^ TAMs. Thus, Tim-4^+^ and Tim-4^–^ TAMs are metabolically programed to have distinct survival capacities and functions in the tumor microenvironment.

Given that Tim-4 protein is rarely expressed in human ovarian cancer–associated macrophages, Tim-4 cannot be used to define human counterparts of mouse Tim-4^+^ TAM subsets. CRIg (encoded by *Vsig4*), a macrophage signature marker, is coexpressed with Tim-4 in mouse F4/80^hi^MHC-II^lo^ peritoneal macrophages (21) and Lyve1^hi^MHC-II^lo^ interstitial macrophages (54). We have explored CRIg as a potential marker to define human ovarian cancer macrophage subsets. Similar to mouse Tim-4^+^ TAMs, transcriptome and functional analysis reveals enriched lysosome-related genes, OXPHOS pathway, and autophagy pathways in CRIg^+^ TAMs in human ovarian cancer. The data suggest that CRIg can be an operational marker to study TAM subsets in human ovarian cancer. In conclusion, based on Tim-4 (or CRIg in humans), we have identified 2 ontogenically, phenotypically, metabolically, and functionally distinct peritoneal macrophage subsets in ovarian cancer (see graphical abstract). We suggest that specifically targeting human CRIg^+^ TAMs may be a meaningful approach for treating peritoneal cancer metastasis.

## Methods

### Mouse models.

Female 6- to 8-week-old *Cd45.2* C57BL/6 mice (Jackson Laboratory), *Cd45.1* C57BL/6 mice (Jackson Laboratory), *Ccr2*^–/–^ mice (Jackson Laboratory), LysM-Cre C57BL/6 mice (Jackson Laboratory), and floxed FIP200 (*Fip200^fl/fl^*) C57BL/6 mice (58) were used for this study. *Fip200^fl/fl^* mice (“*Fip200^+/+^* mice”) were intercrossed with LysM-Cre mice to delete the *loxP*-flanked FIP200 alleles specifically in myeloid cells (“*Fip200^–/–^* mice”). All mice were bred in-house and maintained in specific pathogen–free conditions. All animal research performed was approved by the IACUC at the University of Michigan. MC38 colon carcinoma cells and ID8 ovarian cancer cells were used for this work. Mouse ovarian cancer cell line ID8 was originally from George Coukos, University of Pennsylvania, Philadelphia, Pennsylvania, USA, and mouse colon cancer cell line MC38 was originally from Walter Storkus, University of Pittsburgh, Pittsburgh, Pennsylvania, USA. ID8-luciferase cells (1.5~2 × 10^6^) were injected into the peritoneal cavity of WT mice. Tumor progression was monitored 1 to 2 times per week by Xenogen IVIS Spectrum in vivo bioluminescence imaging system (PerkinElmer). For NAC treatment, 4-week-old *Fip200^–/–^* tumor-bearing mice were I.P. injected with NAC in PBS at a dose of 150 mg/kg every other day. Two weeks after NAC treatment, apoptosis of TAMs was analyzed. For inhibiting monocyte migration, mice were I.P. injected with 50 μg/kg CCR2 antagonist (sc-202525, Santa Cruz Biotechnology) every other day for 2 weeks. Then pretreated mice were inoculated with 2 × 10^6^ ID8 tumor cells and continued with CCR2 antagonist injection for 6 weeks.

### Human ovarian cancer tissues and ascites.

Sixteen patients with high-grade serous ovarian cancer were recruited for this study. Ascites, omentum metastasis, and ovarian cancer tissues were collected from patients with informed consent according to the procedures approved by the Institutional Review Boards of the University of Michigan School of Medicine and the Henry Ford Health System. We used clinical samples from people who had received no prior anticancer therapies. Fresh tumor tissues and ascites were processed into single-cell suspensions for phenotype and functional studies.

### Isolation of macrophages from the peritoneal cavity.

Peritoneal Tim-4^+^ normal residential macrophages were enriched from 5–8 mL peritoneal elute fluid by PE–anti–Tim-4 antibody (clone RMT4-54, BD Biosciences) and anti-PE microbeads (Miltenyi Biotec). To isolate TAM subsets, most tumor cells and dead cells in peritoneal wash of tumor-bearing mice were first removed via density gradient centrifuge by overlaying 15 mL single-cell suspension on 20 mL of 75% Ficoll above 15 mL 100% Ficoll. After Ficoll, Tim-4^+^ TAMs were isolated by using the PE–anti–Tim-4 antibody and anti-PE microbeads from collected leukocyte mononuclear cells. Then, Tim-4^–^ TAMs were enriched from Tim-4^–^ cells through anti-F4/80 MicroBeads UltraPure (Miltenyi Biotec). Total TAMs were enriched from collected leukocyte mononuclear cells through anti-F4/80 MicroBeads UltraPure (Miltenyi Biotec). For Western blot and quantitative PCR (qPCR) analysis, 5 × 10^5^ fresh purified TAMs were directly used. In certain experiments, 5 × 10^5^ TAMs were seeded in 1 well of a 24-well plate for 2 hours, then treated with autophagy inhibitor chloroquine (MilliporeSigma). Media with/without amino acids were formulated with RPMI1640 (R8999-04A, US Biological) by supplementation or omission of amino acids. The medium was supplemented with 10% (*v/v*) dialyzed FBS. For flow cytometry, TAMs were incubated with 500 μL accutase (Life Technologies, Thermo Fisher Scientific) for 30 minutes at 37°C; then detached TAMs were washed and collected for staining.

### Macrophage depletion and adoptive transfer.

To deplete peritoneal resident macrophages, C57BL/6 mice were treated with 1 dose of clodronate-containing liposomes (100 μL each). Control mice were treated with same volume of control liposomes. After 2 weeks, mice were I.P. implanted with ID8 tumor cells. For competition experiments, donor Tim-4^+^ peritoneal macrophages were isolated from several *Fip200^–/–^* CD45.2 congenic mice and pooled together. Then, 8 × 10^5^ purified cells were immediately admixed with 2 × 10^6^ ID8-luciferase tumor cells and injected I.P. into host CD45.1 mice. The cell death of CD45.1 and CD45.2 TAMs was detected 4 weeks later. To explore monocyte-derived macrophages in ID8 tumor–bearing mice, 1 million monocytes were enriched from bone marrow of CD45.1 mice and I.P. injected into 4-week tumor-bearing mice. Three days later, the phenotype of monocyte-derived TAMs was analyzed by flow cytometry.

### FACS and analysis.

Mouse single-cell suspensions were prepared from peripheral blood and from the peritoneal cavity and blocked with rat anti–mouse CD16/CD32 antibodies (eBioscience, Thermo Fisher Scientific) (1/200) for 10 minutes, pelleted by centrifugation (500*g* for 5 minutes; room temperature), subsequently labeled with fluorophore-conjugated anti-mouse antibodies at recommended dilutions for 30 minutes in a dark room, and washed with staining buffer. To quantitate the cells, 25 μL CountBright absolute counting beads (Thermo Fisher Scientific) were added to the samples. For proliferation assays, mice were injected with BrdU (200 μL/mouse) (Invitrogen, Thermo Fisher Scientific) I.P. 3 hours before sacrifice. Cytofix/Cytoperm kit was used to stain for BrdU (BD Biosciences). For detecting SG_2_M phase, transcription factor staining buffer set (eBioscience, Thermo Fisher Scientific) was used to stain for Ki67. PBS-diluted DAPI (1:1000) was used to stain for the DNA content. To quantitate blood monocytes, 100 μL of blood was obtained and incubated in red blood cell lysis buffer (BD Biosciences) for 10 minutes and stained with fluorophore-conjugated antibodies for 30 minutes in a dark room. For apoptosis, cells were evaluated with an annexin V apoptosis detection kit (BD Biosciences). For autophagy quantification in TAM subsets, FlowCellect Autophagy LC3 Antibody-based Assay Kit (MilliporeSigma) was used. This kit disrupts the cell plasma membrane and extracts cytosolic LC-3 by flushing away during washing steps. LC-3 translocated into the autophagosome is protected from the extraction and remains intact inside autophagosome, thereby allowing its fluorescence to be measured by flow cytometry. Events were processed on LSR II and LSRFortessa flow cytometers (BD Biosciences), and data were analyzed with DIVA software (BD Biosciences). The antibodies used for flow cytometry are listed in Supplemental Table 2.

### Measurement of mitochondria content, mitophagy, and ROS staining.

To detect mitochondria activity of fresh TAMs, we first collected leukocyte mononuclear cells from peritoneal elute cells by density gradient centrifuge. Then, we stained leukocyte mononuclear cells with macrophage surface antibodies for 30 minutes. Following this, cells were washed and stained with mitochondria reagents (Life Technologies, Thermo Fisher Scientific) for 30 minutes at 37°C in RPMI-1640 without FBS. MitoTracker Green (100 nM) and MitoTracker Deep red (100 nM) were combined to detect the mitochondria mass. MitoSOX (5 μM) was used to check mitochondria-related ROS. To detected the effect of arginine on the induced mitophagy, TAMs were treated with oligomycin (10 μM) and antimycin A (4 μM) (control) in the presence or absence of rapamycin (100 nM). Meanwhile, arginine or arginanse-1 inhibitor nor-NOHA (0.5 mM) was added into the culture for 24 hours. The levels of damaged mitochondria without any inhibitors were used as background control. The percentages of accumulated damaged mitochondria were normalized to the control group. After washing with PBS for 2 times, the stained TAMs were gated and detected via BD LSR flow cytometry.

### Extracellular flux analysis.

Analysis of the OCR was performed with a Seahorse XF96 Extracellular Flux Analyzer instrument. Sorted TAM subsets were seeded at 2 × 10^5^ per well (96-well) in RPMI-1640 with 10% FBS and incubated for 1 to 2 hours. The media were removed and replaced with Seahorse assay media with 2 mM glutamine and 25 mM glucose. The plates containing cells were incubated for 1 hour at 37°C without CO_2_. Extracellular flux analysis was performed at 37°C without CO_2_ in the XF96 analyzer (Seahorse Bioscience) following the manufacturer’s instructions. Port additions and times are indicated in the figures. Oligomycin (1.25 μM), FCCP (0.5 μM), and rotenone (1 μM) plus antimycin A (1 μM) were injected where relevant, and OCR (pmol O_2_/min) was measured in real time.

### Arginine uptake and arginase activity assay.

To detect the arginine uptake in TAM subsets, 5 × 10^5^ TAMs were seeded in a 24-well plate for 24 hours. Then, culture media with or without TAMs were collected to detect the arginine amount with the l-arginine assay kit (BioVision). The levels of arginine uptake were determined by using the amount of arginine in culture medium without TAMs to subtract the amount of arginine in culture medium with TAMs. To detect the arginase activity in TAM subsets, 5 × 10^5^ fresh TAMs were lysed in 10 mM Tris-HCl 7.4 buffer containing 0.4% (*w/v*) Triton X-100 and protease inhibitors. The arginase activity was measured by the Arginase Assay Kit (Abnova). The lysed cells were centrifuged at 14,000*g* at 4°C for 10 minutes, and the supernatants were plated onto a 96-well microtiter plate. l-arginine was converted to urea by a buffer containing a substrate and cofactor, and the absorbance of the samples was measured using a microplate reader at the wavelength of 430 nm.

### Real-time PCR and RNA-Seq analysis.

Total RNA was isolated from cells by column purification (Direct-zol RNA Miniprep Kit, Zymo Research) with DNase treatment. cDNA was synthesized using High-Capacity cDNA Reverse Transcription Kit (Thermo Fisher Scientific) with poly-dT or random hexamer primers. qPCR was performed on cDNA using Fast SYBR Green Master Mix (Thermo Fisher Scientific) on a StepOnePlus Real-Time PCR System (Thermo Fisher Scientific). Fold changes in mRNA expression were calculated by the ΔΔCt method using *Actb* as an endogenous control. Results are expressed as fold change by normalizing to the controls. The primers used for qPCR are listed in Supplemental Table 2. The acquisition and analysis of RNA-Seq data were described previously (59). Total RNA was isolated from cells by column purification (Direct-zol RNA Miniprep Kit, Zymo Research) with DNase treatment. The Ribo-Zero Gold rRNA Removal Kit (Illumina) and TruSeq Stranded Total RNA Library Prep Globin kit (Illumina) were used to prepare the library for RNA-Seq. Sequencing was performed by the University of Michigan DNA Sequencing Core, using the Illumina Hi-Seq 4000 platform, paired end, 50 cycles. Quality of the raw reads data for each sample was first evaluated using FastQC (version 0.11.3). The Tuxedo Suite software package was used for alignment, differential expression analysis, and postanalysis diagnostics. In brief, reads were aligned to the reference transcriptome (hg19) using TopHat (version 2.0.13), and a second round of quality control was performed after alignment. Cufflinks/CuffDiff (version 2.2.1) was used for expression quantification, normalization, and differential expression analysis. Locally developed scripts were used to format and annotate the differential expression data output from CuffDiff. Diagnostic plots were generated using the cummeRbund R package. Data were deposited in the NCBI’s Gene Expression Omnibus database (GSE157673)

### Western blot.

Cells were dissolved in RIPA Lysis and Extraction Buffer (Thermo Fisher Scientific) supplemented with protease inhibitor cocktail (Roche). The protein concentrations were determined by Bio-Rad protein assay reagent. The lysates were boiled for 5 minutes in 3× SDS sample buffer (0.5 M Tris-HCl pH 6.8, 30% glycerol, 3% SDS, 0.01% bromphenol blue) containing 3% β-mercaptoethanol and were analyzed by SDS-PAGE followed by Western blot using different antibodies, including arginase-1 (93668, Cell Signaling Technology), arginase-2 (55003, Cell Signaling Technology), CDK4 (11026-1-AP, Proteintech), CDK6 (3136, Cell Signaling Technology), cleaved caspase-3 (Asp175) (9661, Cell Signaling Technology), COX IV (4844, Cell Signaling Technology), cyclin D1 (2922, Cell Signaling Technology), cyclin D3 (2936, Cell Signaling Technology), FIP200 (17250-1-AP, Proteintech), LC3A/B (12741, Cell Signaling Technology), phospho-histone H2A.X (Ser139) (9718, Cell Signaling Technology), phospho-p70 S6 kinase (Thr389) (9205, Cell Signaling Technology), phospho-S6 (Ser235/236) (4858, Cell Signaling Technology), phospho-ULK1 (Ser317) (12753, Cell Signaling Technology), phospho-ULK1 (Ser757) (14202, Cell Signaling Technology), SDHB (ab14714, Abcam), SQSTM1/p62 (5114, Cell Signaling Technology), ULK1 (8054, Cell Signaling Technology), and β-actin (A5441, MilliporeSigma). Signals were detected by ECL reagents (Thermo Fisher Scientific). The protein expression levels were quantified with ImageJ (NIH) software and were normalized to 1 in specific control groups.

### Enrichment and gene ontology analysis.

GSEA was performed using the GSEA software downloaded from Broad Institute (60). The gene signatures for GSEA are listed in Supplemental Table 2. The function was used to compute the enrichment scores and simulated enrichment scores for each variable and signature. A comprehensive GSEA web server, Enrichr (61), was used for Kyoto Encyclopedia of Genes and Genomes (KEGG) pathway and gene ontology analysis in human TAMs. Important KEGG pathways and gene ontology terms were selected.

### Statistics.

Mann-Whitney *U* test was used to compare 2 independent groups. Student’s *2-tailed* *t* test was used for paired samples. The Pearson correlation was used to analyze the association between 2 continuous variables. For multigroup comparisons, 1-way ANOVA with Dunnett’s multiple-comparisons test was used to identify group-specific differences. Statistical analysis was performed with GraphPad Prism 8 software (GraphPad Software, Inc.). *P* < 0.05 was considered significant.

### Study approval.

All patients provided informed written consent. All human samples were obtained in accordance with the following protocols approved by the Institutional Review Boards of the University of Michigan School of Medicine and the Henry Ford Health System (HUM00035663). All murine studies were performed under approval and in accordance with IACUC at the University of Michigan protocols (PRO00008278).

## Author contributions

HX, IK, and WZ conceptualized the study; HX, IK, Weichao Wang, YB, SW, SG, Weimin Wang, LV, and JLG determined methodology and performed animal studies; XL and SL performed statistical and bioinformatics analysis; JRL, KM, RR, and AM performed pathological and clinical studies; HX and WZ wrote the original draft; WZ acquired funding; and IK and WZ supervised the study.

## Supplementary Material

Supplemental data

Supplemental Table 1

Supplemental Table 2

## Version 1 08/11/2020

In-Press Preview

## Version 2 09/17/2020

Electronic publication
