# Supplementary material for: Autophagic adaptation to oxidative stress alters peritoneal residential macrophage survival and ovarian cancer metastasis
Source: JCI Insight. 2020 Sep 17;5(18):e141115. doi: 10.1172/jci.insight.141115 (PMC7526547; doi:10.1172/jci.insight.141115)
Supplement: Supplemental data [file jciinsight-5-141115-s120.pdf]

Supplemental data

Supplemental Figure 1.

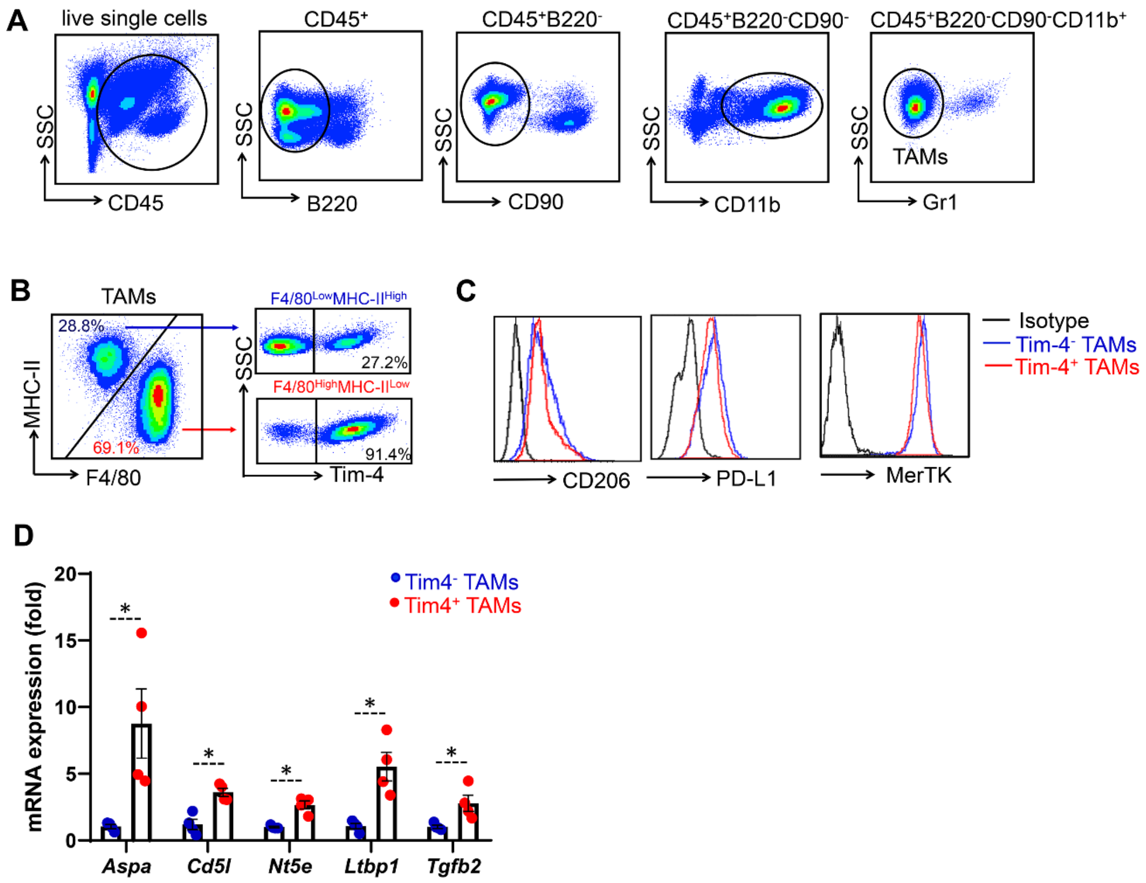

Supplemental Figure 1. Tim-4 defines two distinct peritoneal macrophage subsets in ovarian cancer. (**A**) Representative flow cytometry plots showing the gating strategy to define peritoneal TAMs in mice bearing peritoneal ID8 ovarian cancer. (**B**) Relationship between Tim-4^{+} TAMs and F4/80 and MHC-II expressing TAMs. One of 10 is shown. (**C**) Expression of CD206, PD-L1, and MerTK on Tim-4^{+} and Tim-4^{-} TAMs. One representative of 3 is shown. (**D**) mRNA expression of GATA6 down-stream genes in Tim-4^{+} and Tim-4^{-} TAMs (n = 4 mice/group, mean ± SEM) * $P < 0.05$ (Mann-Whitney U tests).

Supplemental Figure 2.

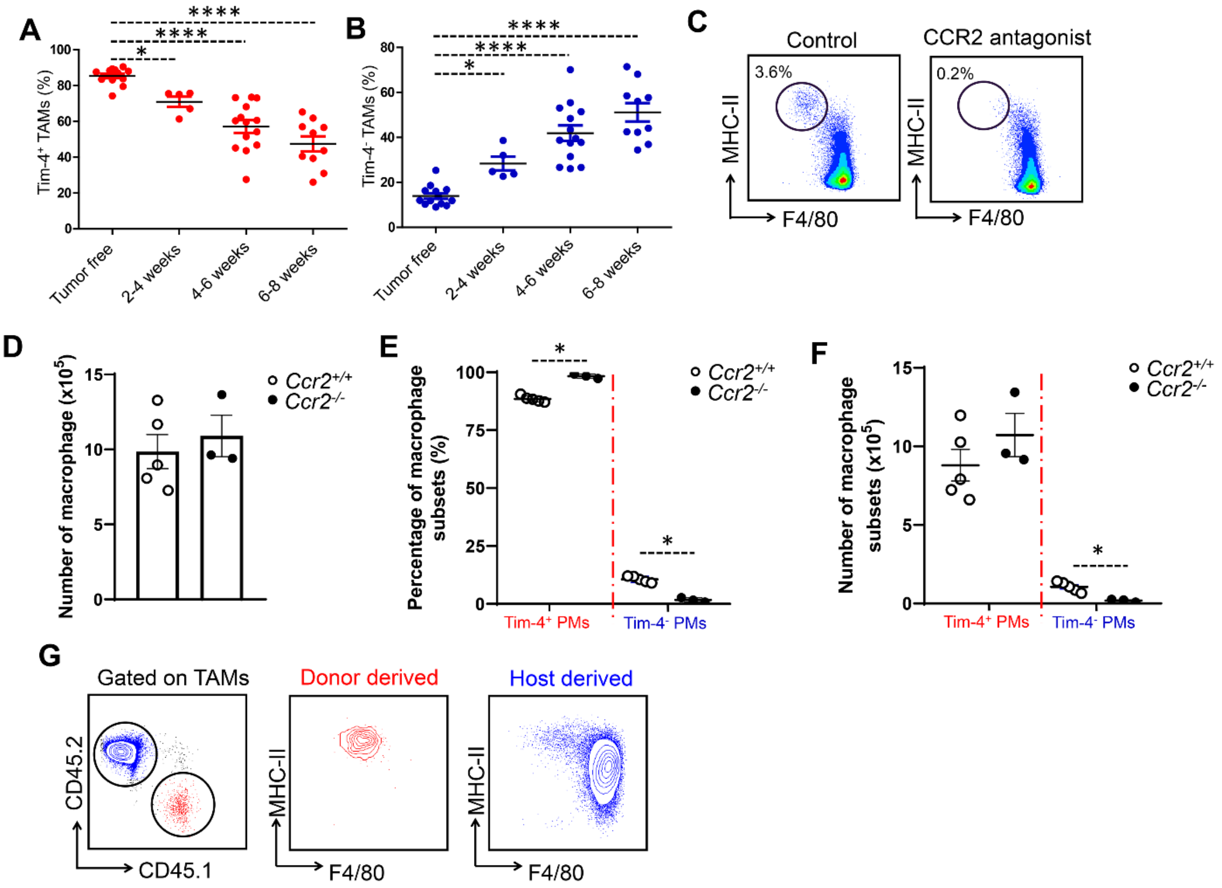

Supplemental Figure 2. Tim-4⁻ TAMs migrate from peripheral monocytes without affecting tumor growth. (**A and B**) Dynamic changes of Tim-4⁺ (**A**) and Tim-4⁻ peritoneal TAMs (**B**) during ovarian cancer progression. TAMs were analyzed by FACS at different time points. (n = 5-14 mice/group, mean ± SEM). * P <0.05, **** P < 0.0001(one-way ANOVA with Dunnett's multiple comparisons test) between normal mice and mice bearing ID8 tumor at Week 2-4, 4-6, and 6-8. (**C**) Effect of CCR2 antagonist on peritoneal macrophages in tumor free mice (n = 5). (**D**) Number of peritoneal macrophage in wild type and *Ccr2*$^{-/-}$ tumor free mice (n = 3-5 mice/group, mean ± SEM). P > 0.05. (**E and F**) Percentage (**E**) and number (**F**) of Tim-4⁺ and Tim-4⁻ peritoneal macrophages (PMs) in wild type and *Ccr2*$^{-/-}$ tumor free mice (n = 3-5 mice/group, mean ± SEM). * P <0.05 (Mann-Whitney U tests). (**G**) Representative flow cytometry plots of the monocyte derived TAMs. CD45.1⁺ monocytes were transferred into ID8 tumor-bearing mice. CD45.1⁺ monocyte derived TAMs (red) were analyzed with F4/80 and MHC-II expression. One representative of 4 is shown.

Supplemental Figure 3.

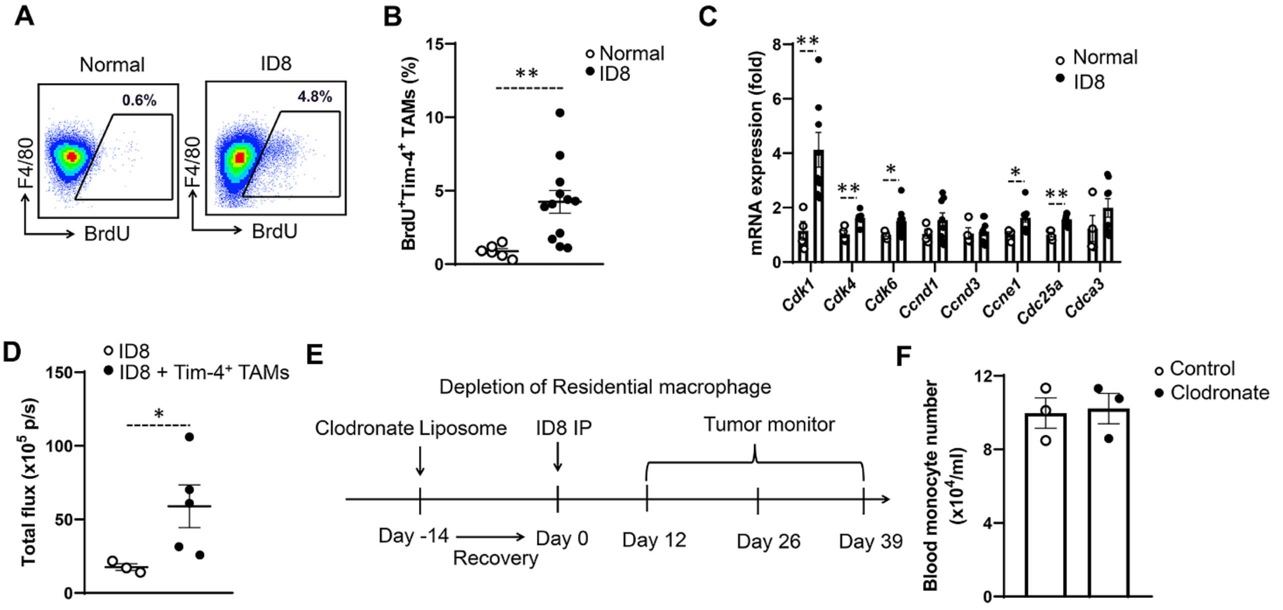

Supplemental Figure 3. Tim-4$^+$ TAMs are embryonically derived proliferative cells with pro-tumor function. (**A and B**) Representative flow cytometry showing the percentages of BrdU$^+$ (**A**) and BrdU$^+$Tim-4$^+$ (**B**) peritoneal residential macrophages. Peritoneal macrophages were from tumor-free and ID8 tumor bearing mice (*n* = 6-12 mice/group, mean ± SEM). ** *P* <0.01 (Mann-Whitney *U* tests). (**C**) mRNA expression of cell cycle related genes in Tim-4$^+$ TAMs normalized to Tim-4$^+$ peritoneal residential macrophage (*n* = 4-8 mice/group, mean ± SEM). ** *P* <0.01 (Mann-Whitney *U* tests) on the expression of *Cdk1, Cdk4, Cdc25a*. * *P* <0.05 (Mann-Whitney *U* tests) on the expression of *Cdk6, Ccne1*. (**D**) Effect of Tim-4$^+$ TAMs on ID8 growth. Wild type mice were inoculated (I.P) with ID8 cells or ID8 cells plus Tim-4$^+$ TAMs. Tumor volume was shown at day 27 (*n* = 3-5 mice/group, mean ± SEM). * *P* < 0.05 (Mann-Whitney *U* tests). (**E**) Scheme of experimental procedure, including peritoneal residential macrophage depletion and tumor inoculation. (**F**) Number of blood monocytes in tumor free mice treated with control liposome or clodronate liposome, *P* > 0.05 (*n* = 3 mice/group, mean ± SEM).

Supplemental Figure 4.

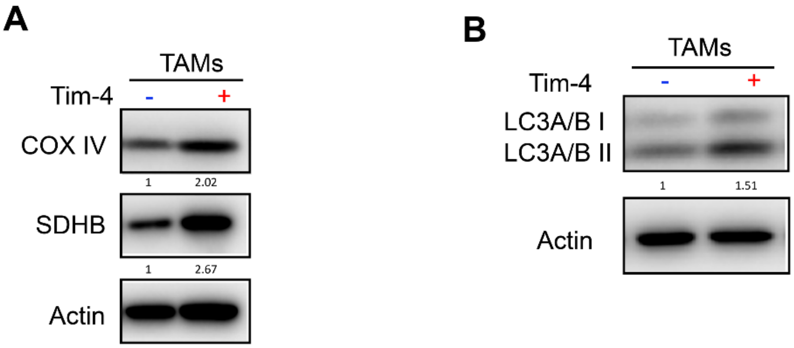

Supplemental Figure 4. Tim-4$^+$ TAMs exhibit and maintain high mitochondria activity and autophagy function. (**A**) Expression of mitochondria related proteins in Tim-4$^+$ and Tim-4$^-$ TAMs. One representative of 3 is shown. (**B**) LC-3 in Tim-4$^+$ and Tim-4$^-$ TAMs. One representative of 3 is shown.

Supplemental Figure 5.

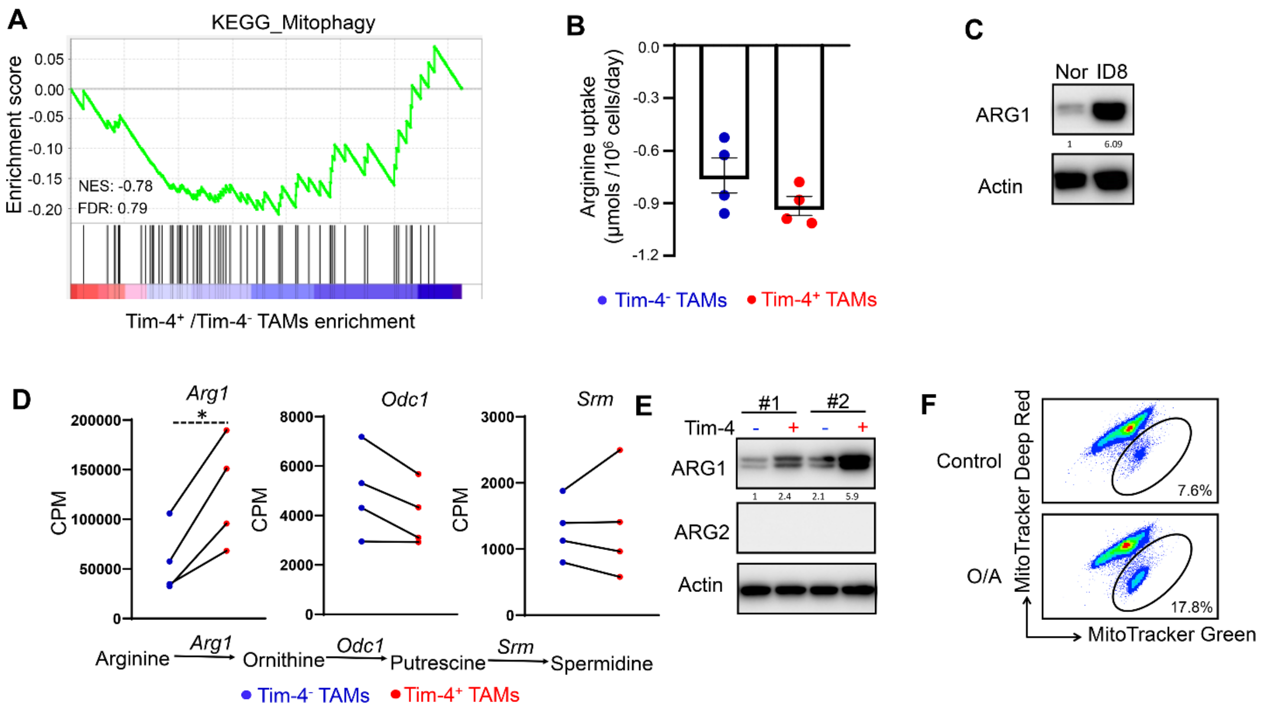

Supplemental Figure 5. Arginase-1 impacts mitochondria fitness and mitophagy via mTORC1 in Tim-4⁺ TAMs. (**A**) Enrichment of mitophagy gene set in Tim-4⁺ TAMs compared with Tim-4⁻ TAMs. Normalized enrichment score (NES), false discovery rate (FDR). FDR < 0.25 is considered significant. (**B**) Measurement of arginine uptake in Tim-4⁻ TAMs and Tim-4⁺ TAMs. $P > 0.05$ ($n = 4$ mice/group, mean ± SEM). (**C**) Western blot showing arginase-1 expression in Tim-4⁺ PMs and Tim-4⁺ TAMs. One representative of 3 is shown. (**D**) Expression of *Arg1*, *Odc1*, and *Srm* in Tim-4⁻ TAMs and Tim-4⁺ TAMs based on RNA sequencing data. The paired Tim-4⁺ and Tim-4⁻ TAMs were from same mice. ($n = 4$ mice/group). * $P < 0.05$ (Mann-Whitney *U* tests). (**E**) Western blot showing arginase 1 and arginase 2 expression in Tim-4⁻ TAMs and Tim-4⁺ TAMs. One representative of 3 is shown. (**F**) Representative flow cytometry plots showing damaged mitochondria in Tim-4⁺ TAMs treated with or without oligomycin (10 μM) plus antimycin A (4 μM) for 24 hours. One of 4 is shown.

Supplemental Figure 6.

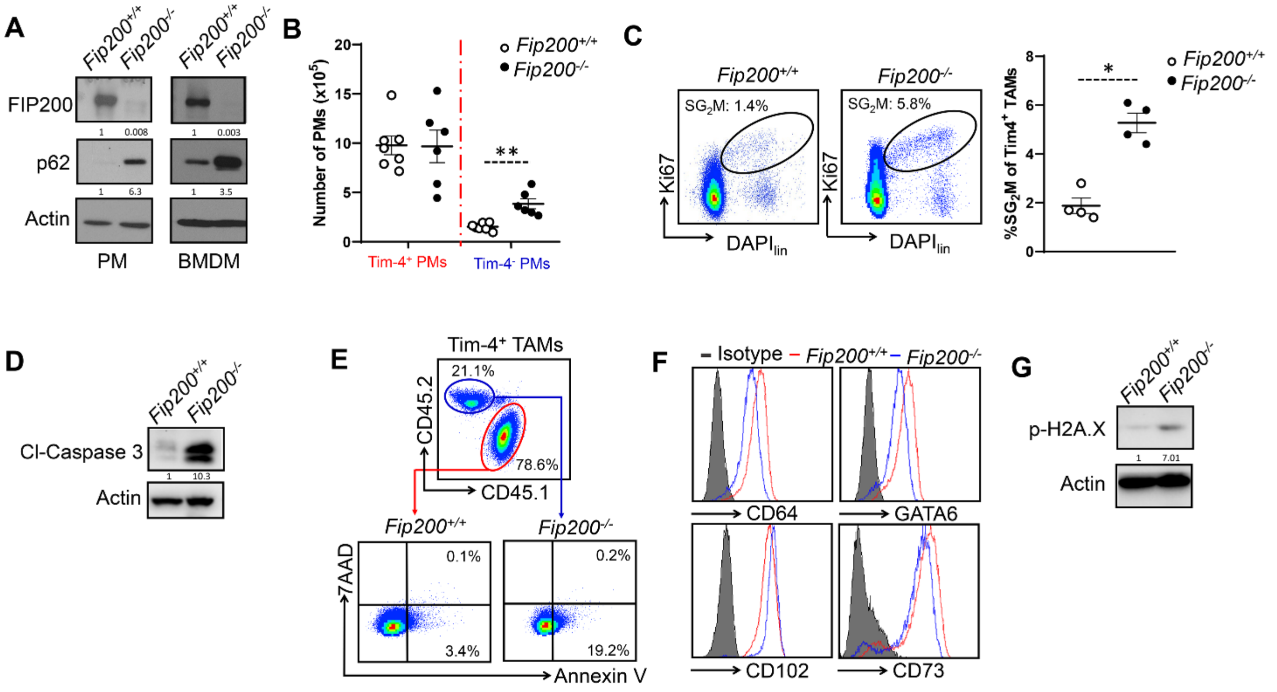

Supplemental Figure 6. Autophagy deficiency results in loss of Tim-4⁺ TAMs in ovarian cancer. (**A**) FIP200 and p62 protein expression in Tim-4⁺ peritoneal macrophage (PM) and bone marrow derived macrophage (BMDM) from *Fip200*$^{+/+}$ and *Fip200*$^{-/-}$ tumor-free mice. One of 3 is shown. (**B**) Effect of FIP200 deficiency on mouse peritoneal macrophage (PM) subsets. Tim-4 expression was determined by FACS (*n* = 6 to 7 mice/group, mean ± SEM). ** *P* <0.01 (Mann-Whitney *U* tests) for Tim-4^{-} PMs between *Fip200*$^{+/+}$ and *Fip200*$^{-/-}$ tumor-free mice. (**C**) Percentage of Tim-4^{+} TAMs with self-renewal in *Fip200*$^{+/+}$ and *Fip200*$^{-/-}$ ID8 tumor bearing mice (*n* =4 mice/group mice/group, mean ± SEM). Numbers on the dot plots represent the percentage of Tim-4^{+} TAMs in SG_2M phase. * *P* < 0.05 (Mann-Whitney *U* tests). (**D**) Western blot showing cleaved caspase 3 expression in Tim-4^{+} TAMs from *Fip200*$^{+/+}$ and *Fip200*$^{-/-}$ ID8 tumor bearing mice. One representative of 3 is shown. (**E**) Role of FIP200 deficiency on Tim-4^{+} TAM apoptosis. Normal CD45.2^{+}Tim-4^{+}FIP200$^{-/-}$ peritoneal macrophages were inoculated with ID8 tumor cells into peritoneal cavity in CD45.1^{+} wild type mice. CD45.1 and CD45.2 were used to define host and donor Tim-4^{+} TAMs, respectively. Cell apoptosis was analyzed with Annexin V and 7-AAD staining. Numbers on the dot plots represent the percentage of Annexin V^{+}Tim-4^{+} TAMs. One representative of 5 is shown. (**F**) Phenotype of Tim-4^{+} TAMs in *Fip200*$^{+/+}$ and *Fip200*$^{-/-}$ ID8 tumor bearing mice. The phenotype of TAMs was determined by FACS. Gates were on peritoneal Tim-4^{+} TAMs. One representative of 3 is shown. (**G**) Expression of p-H2A.X in Tim-4^{+} TAMs between *Fip200*$^{+/+}$ and *Fip200*$^{-/-}$ ID8 tumor bearing mice. One representative of 3 is shown.

Supplemental Figure 7.

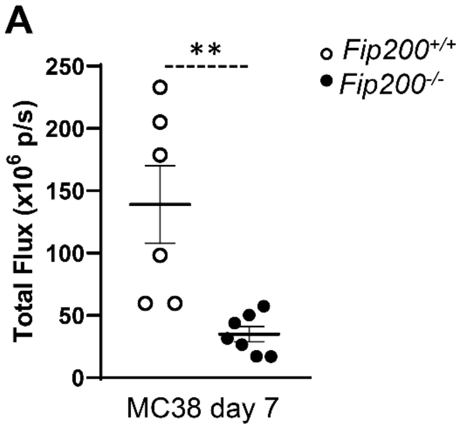

Supplemental Figure 7. Autophagy deficiency in macrophages supports T cell-mediated anti-tumor immunity. (**A**) Effect of macrophage FIP200 deficiency on peritoneal colon cancer growth. Wild type (*Fip200*$^{+/+}$) and FIP200 deficient (*Fip200*$^{-/-}$) mice were inoculated (I.P) with luciferase expressing MC38 colon tumor cells. Tumor volume was monitored. (*n* = 6 to 7 mice/group, mean ± SEM) ** *P* < 0.01 (Mann-Whitney *U* tests).

Supplemental Figure 8.

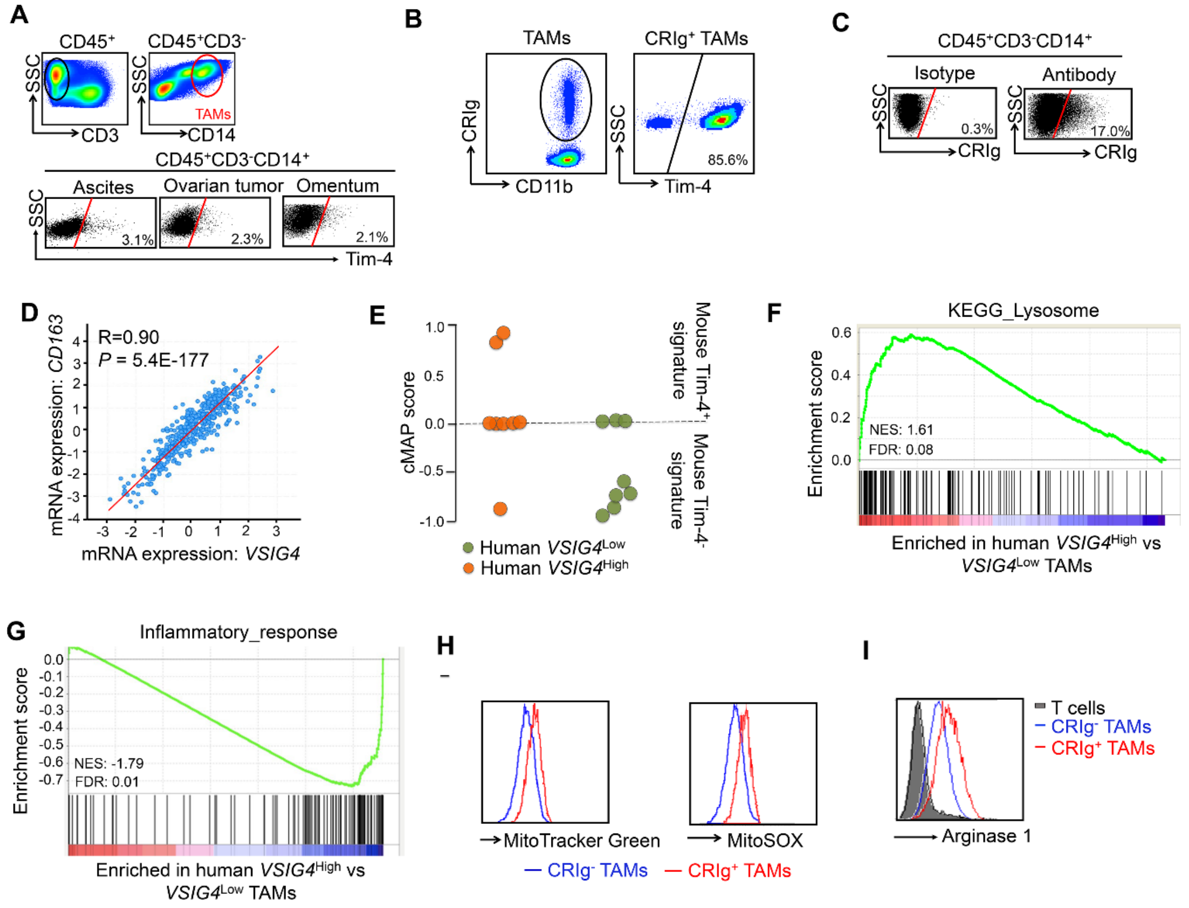

Supplemental Figure 8. CRIg$^+$ TAMs are the murine counterparts of Tim-4$^+$ TAMs in human ovarian cancer. (**A**) Tim-4 expression on human TAMs. The surface expression of Tim-4 was analyzed by FACS on CD45$^+$CD3$^-$CD14$^+$ TAMs from ascites, ovarian cancer tissues, and ovarian cancer associated omentum. One representative of 3 is shown. (**B**) Relationship between Tim-4 and CRIg expression on mouse TAMs. Expression of Tim-4 and CRIg was analyzed by FACS on peritoneal TAMs in ID8 tumor bearing mice. One representative of 4 is shown. (**C**) CRIg expression on human TAMs. The surface staining of CRIg on human TAMs from ovarian cancer associated omentum in patients with ovarian cancer. One representative of 3 is shown. (**D**) Scatterplot showing Pearson's correlation between *CD163* and *VSIG4* expression in human high grade serous ovarian serous carcinoma. Red line indicates regression fit. (**E**) cMap analysis of human ovarian cancer related *VSIG4*^{High} and *VSIG4*^{Low} TAMs showing their enrichment for mouse Tim-4$^+$ and Tim-4$^-$ TAMs gene sets established in (Figure 1E). (**F**) Enrichment of lysosome gene set in *VSIG4*^{High} TAMs compared with *VSIG4*^{Low} TAMs. Normalized enrichment score (NES), false discovery rate (FDR). FDR < 0.25 is considered significant. (**G**) Enrichment of inflammatory response gene set in *VSIG4*^{Low} TAMs compared with *VSIG4*^{High} TAMs. Normalized enrichment score (NES), false discovery rate (FDR). FDR < 0.25 is considered significant. (**H**) Mitochondrial mass and mitochondria related ROS in human ovarian CRIg$^+$ and CRIg$^-$ TAMs. One representative of 3 is shown. (**I**) Arginase-1 expression in human ovarian CRIg$^+$ and CRIg$^-$ TAMs. One representative of 3 is shown.

Graphical Abstract

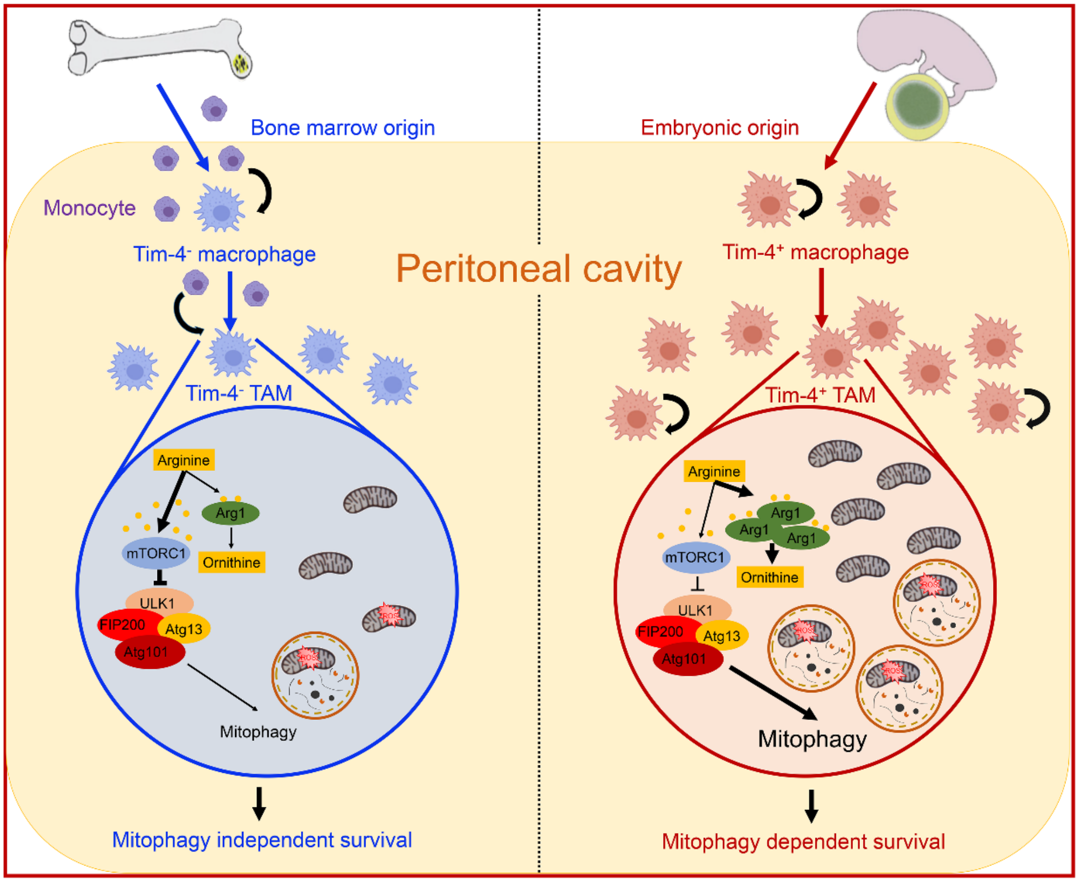

Graphical abstract

Tim-4$^+$ and Tim4$^-$ TAMs in ovarian cancer-associated peritoneal metastasis. Tim-4$^+$ TAMs were embryonically originated and locally sustained, and promoted tumor progression; while Tim-4$^-$ TAMs replenished from circulating monocytes. Relative to Tim-4$^-$ TAMs, Tim-4$^+$ TAMs expressed high level of mitochondria activity and ROS production, which forced them adapt to mitophagy to alleviate oxidative stress. High arginase-1 in Tim-4$^+$ TAMs supported mitophagy formation via weakened mTORC1 activation due to low arginine resultant from arginase-1-mediated arginine metabolism. Tim-4$^+$ TAMs, but not Tim-4$^-$ TAMs, depended on mitophagy to survive in the tumor microenvironment. Autophagic adaptation to oxidative stress alters peritoneal residential macrophage phenotype and ovarian cancer progression

Supplementary Table 1. Analysis of RNA-Sequencing data related to mouse TAM subsets

Supplementary Table 2. Lists of gene signatures, flow antibodies and primers
